# Nicotinic acetylcholine receptors: *Ex-vivo* expression of functional, non-hybrid, heteropentameric receptors from a marine arthropod, *Lepeophtheirus salmonis*

**DOI:** 10.1371/journal.ppat.1008715

**Published:** 2020-07-27

**Authors:** Lucien Rufener, Kiranpreet Kaur, Anouk Sarr, Stian Mørch Aaen, Tor Einar Horsberg

**Affiliations:** 1 INVENesis Sarl, chemin de Belleroche, Neuchâtel, Switzerland; 2 Norwegian University of Life Sciences, Faculty of Veterinary Medicine, Sea Lice Research Centre, Oslo, Norway; 3 Aker BioMarine AS, Lysaker, Norway; 4 Aqua Pharma Group, Hovemoveien, Lillehammer, Norway; McGill University, CANADA

## Abstract

Nicotinic acetylcholine receptors (nAChRs) are ligand-gated ion channels mostly located in the post-synaptic membrane of cholinergic synapses. The natural neurotransmitter is acetylcholine, but they are also the direct targets for neonicotinoids, chemicals widely used against ectoparasites, arthropod vectors and agricultural pests. There are significant concerns regarding adverse effects of neonicotinoids on beneficial insects. In arthropods, functional nAChRs made of α subunits have been expressed from *Drosophila* genes, and hybrid receptors (sometimes also referred to as chimeric receptors) using species-specific α subunits and vertebrate β subunits have been expressed *ex-vivo*. Arthropod-specific nAChRs made of both α and β subunits from the target species have not been expressed *ex-vivo*. The aim of the current study was to express such receptors in *Xenopus* oocytes using only genes from *Lepeophtheirus salmonis*, to characterize them and study their modulation. Genes encoding α and β subunits of the nAChRs and three ancillary proteins, RIC-3, UNC-50 and UNC-74 were identified in the *L*. *salmonis* genome, subjected to RACE-PCR, cloned into an expression vector and the cRNA produced was then injected into *Xenopus laevis* oocytes. Co-expression of the ancillary proteins was essential for the successful expression of the *L*. *salmonis* nAChRs with both α and β subunits. Two functional nAChRs were identified: Lsa-nAChR1 consisting of α1, α2, β1 and β2 subunits, reconstituted to one distinct receptor, while Lsa-nAChR2, consisting of α3, β1 and β2 subunits reconstitutes receptors with two distinct characteristics. Out of seven neonicotinoids tested, six worked as partial agonist of Lsa-nAChR1 while only three did so for Lsa-nAChR2. Four non-neonicotinoid compounds tested had no effect on either of the nAChRs. The study demonstrated that fully functional, non-hybrid nAChRs containing both α and β subunits from an arthropod can be reconstituted *ex-vivo* by co-expression of essential ancillary proteins. Such models would be valuable for in-depth studies of effects by neonicotinoids and other compounds on target pests, as well as for studies of adverse effects on non-target arthropods.

## Author summary

Nicotinic acetylcholine receptors, nAChRs, respond to the neurotransmitter acetylcholine or drugs like nicotine. These receptors are targets for neonicotinoids, the most commonly used compounds against ectoparasites and agricultural pests. In-depth studies of the function of these channels in arthropods are sparse, as no groups managed to reconstitute functional nAChRs made of both α and β subunits using genes only from the target arthropod in an *ex-vivo* system. We report the successful assembly of non-hybrid, fully functional nAChRs containing both α and β subunits from a marine arthropod, assembled and expressed in *Xenopus laevis* oocytes. We identified two possible combinations of α and β subunits producing functional receptors. We found ancillary proteins to be essential for successful expression and assembly of both α and β subunits into a functional receptor. The findings of the present study provide a basis for studying native nAChRs from arthropods, with a switch from hybrid nAChRs to species-specific native nAChRs.

## Introduction

Nicotinic acetylcholine receptors (nAChRs) are members of the Cys-loop ligand gated ion channel superfamily that mediates the transduction of chemoelectric signals in the nervous system and at the neuromuscular junction [1, 2]. Functional nAChRs are homo-or hetero-pentameric complexes, consisting of five homologous subunits arranged around a central ion channel [2]. Each subunit possesses an N-terminal extracellular domain with a Cys-loop, two sulfide bond-forming cysteines separated by 13 amino acid residues. Each subunit has an extensive extracellular N-terminal domain, four transmembrane segments (TM1-TM4), a cytoplasmic loop of variable length and amino acid sequence between the third and fourth transmembrane segments, and a short extracellular C-terminus [3]. Domain TM2 donates most of the residues that line the ion channel, and an intracellular loop between TM3 and TM4 that is involved in receptor localization and modulation of function [3]. The acetylcholine (ACh) binding site is located at the interface of two adjacent subunits and is formed by six distinct regions (loop A-F) in the N-terminal extracellular domain with each of the adjacent subunits contributing three loops [4]. Subunits possessing two adjacent cysteine residues in loop C, which have been shown to be important for ACh binding, are referred to as alpha (α) subunits, whereas subunits lacking these vicinal cysteins are classified as non-alpha (β, γ, δ and ε) subunits [3]. In vertebrates, 17 different subunits have been identified (α1-α10, β1-β4, γ, δ and ε). Distinct nAChRs subtypes, assembled from unique subunit combinations, exhibit different pharmacological characteristics, and physiological functions [1, 2, 5, 6].

Binding of endogenous ACh or exogenous agonists like nicotine to nAChR opens an intrinsic ion channel in the receptor, allowing the flow of cations (mainly Na^+^ and K^+^ but some subunit combinations are also permeable to Ca^2+^) through the cell membrane, inducing a wide variety of biological responses [4, 7]. Mammalian nAChRs are potential targets for several therapeutic approaches, including the treatment of nicotine addiction, Alzheimer’s disease, schizophrenia, and Parkinson’s disease as well as the control of pain [8, 9]. Because of this, these receptors are major targets for pharmaceutical drug discovery and thus their functional assembly and gating mechanisms have been extensively investigated. nAChRs are also expressed extensively in insects and other invertebrates and are potential targets for parasiticides and pesticides [10–12]. Commercially important insecticides, neonicotinoids, are deployed worldwide for the management of various ectoparasites and agricultural pests. These drugs are selectively toxic to invertebrates over vertebrates, which is due to the selectivity of these drugs to nAChRs in invertebrates [10, 13]. Great concerns have been raised regarding the effect of these insecticides on non-target organisms, especially pollinators like honeybees. In addition to direct toxic effects, behavioral changes like impaired navigation abilities and improper development of larvae have been described (reviewed in [14]). However, the limited knowledge of the structure and assembly of native nAChRs in invertebrates has posed a big limitation in our understanding of the interaction of neonicotinoids with nAChRs in arthropods [11, 15].

Insect nAChRs are among the most thoroughly studied of the invertebrate receptors, mainly because of the agricultural and pathological importance [10, 11, 14]. Insect nAChRs exhibit characteristics which are different from those of vertebrates. Insects have fewer nAChR subunits (10–12 subunits discovered so far) compared with mammals (17 subunits) [2]. Although several nucleotide sequences of arthropod nAChR subunits have been identified, and genome sequencing of several insects gives information about insect nAChRs, no functional nAChRs consisting of only arthropod α and β subunits have been obtained in an *ex vivo* system. Functional nAChRs have been obtained by combining only α subunits from *Drosophila melanogaster* in a *Xenopus laevis* oocyte expression system, using the molecular chaperone RIC-3 [16]. The difficulty of combining both α and β subunits in this expression system has hampered the complete understanding of the function of these channels in any arthropod. Attempts have been made to microinject the insect membrane into *Xenopus laevis* oocytes to gain access to native nAChRs embedded in membrane by stimulating the injected oocytes with nicotine [17]. Besides, artificial subunit hybrid receptors (sometimes also called chimeric receptors), with recombinant nAChRs of insect α subunits and vertebrate β subunits have been reconstituted in *Xenopus laevis* oocytes [18–20]. Although these methods are useful strategies to investigate the pharmacological properties of nAChR in insects, they may not faithfully reveal all features of the insect nAChRs. Hence, we are still be missing important knowledge about the native structure, subunit stoichiometry and response to xenobiotics of arthropod nAChRs. Such functional channels would serve as important research tools for physiological and toxicological studies, as well as screening tools for existing and novel compounds targeting nAChRs with specificity towards parasites and pest organisms only. In a review from 2020, the establishment of a functional expression system for insect nAChRs consisting purely of insect subunits was considered particularly urgent [21].

The current study aimed to fill this crucial void in the existing literature by obtaining the successful assembly of non-hybrid, fully functional nAChRs using only α and β subunits from the target arthropod. The model used in the study was an aquatic invertebrate, *Lepeophtheirus salmonis*, commonly known as the salmon louse. This crustacean is an ectoparasite of Atlantic salmon and poses one of the biggest threats to salmon industry across the North Atlantic [22]. Studies with the aim to develop the neonicotinoid imidacloprid as a treatment against salmon lice are ongoing [23]. However, like other arthropods, the full understanding of the mode of action of these compounds is missing in salmon louse.

## Results

### Identification and characterization of eight full-length cDNAs, encoding nAChR subunits from *L*. *salmonis*

We identified eight potential sequences encoding nAChR subunits in *L*. *salmonis* genome by a BLAST algorithm (BLASTp) using conserved sequences from nAChRs in different arthropods. The full-length predicted cDNA sequence could not be identified for two sequences. However, when we compared the expression levels of these transcripts from an RNAseq study (S1 Table) of adult female *L*. *salmonis*, these two sequences had a low expression level. Consequently, we decided to proceed with only the six full-length sequences for this study. The open reading frame, lengths of deduced protein and GenBank accession numbers for these six subunits are provided in the S2 Table.

All these six sequences share substantial sequence similarity with known nAChR subunits from other arthropods. Multiple sequence alignment of the six *L*. *salmonis* nAChR subunit protein sequences along with *Drosophila melanogaster* α1 nAChR sequence (S1 Fig) showed many elements typical of Cys-loop ligand gated ion channels. These include a) an N-terminal signal peptide sequence, b) an extracellular N-terminal region with conserved residues within loops A-F that are involved in ACh binding, c) the di-cysteine (Cys) loop consisting of two di-sulphide-bond forming cysteines separated by 13 amino-acid residues, d) four well-conserved transmembrane regions (TM1-4) and e) a highly variable intracellular loop between TM3 and TM4. In four of the candidate subunits, two adjacent cysteine residues are present in loop C of the ACh-binding site, defining them as α subunits. Due to the absence of these vicinal cysteines, the remaining two candidate subunits were designated as β subunits.

**Fig 1:**
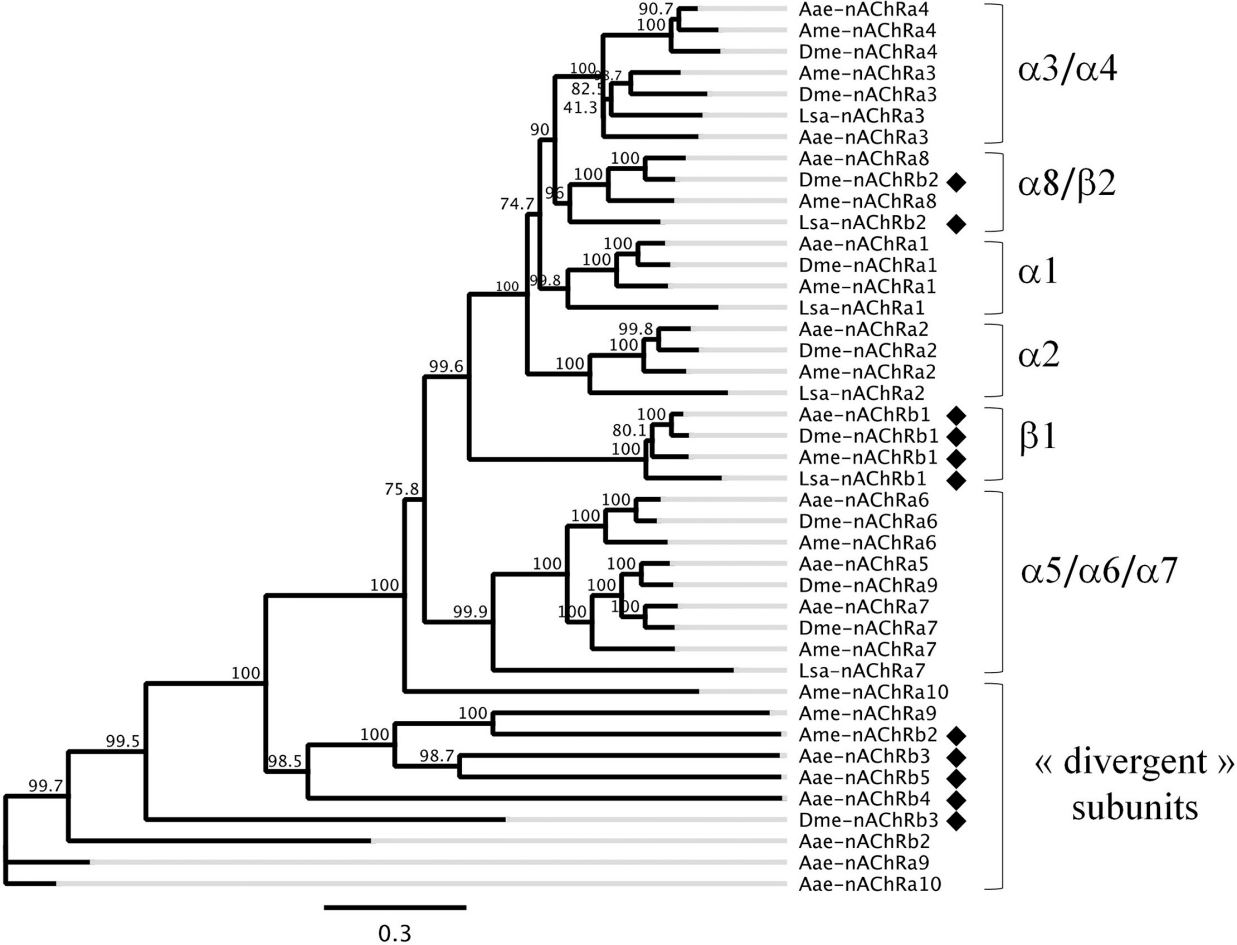
Phylogenetic relations of *L*. *salmonis* nAChR subunits. Consensus phylogram constructed using full-length nAChRs subunits (amino acid sequences) from *Lepeophtheirus salmonis* (Lsa), *Apis melifera* (Ama), *Drosophila melanogaster* (Dme) and *Aedes aegypti* (Aae). Branch labels indicate the consensus support in percent. The tree was constructed using a maximum likelihood approach (PhyML 3.0) and a bootstrap with 1000 iterations. The scale bar represents 0.3 substitutions per site. Black diamonds indicate β subunits.

To understand the evolutionary relationships between the putative nAChR subunits from *L*. *salmonis* with other insects and arthropods, and to obtain a proper nomenclature for these *L*. *salmonis* nAChR subunits, a phylogenetic analysis was performed using these six nAChR sequences and sequences for different nAChR subunits in other arthropods. Based on the previous classification derived from sequence similarity to the *Drosophila melanogaster* subunits, nAChR subunits from different arthropods could be divided into five core groups (α1s, α2s, α3s, α5-α7s and β1s) and two divergent groups. The nAChR subunits in *L*. *salmonis* were named based on their respective cluster groups, e.g., among the six subunits, one sequence clustered with α1s, and therefore named Lsa-nAChRα1. Similarly, another sequence was most closely related to α2s and subsequently named Lsa-nAChRα2. Three other sequences were named as Lsa-nAChRα3, Lsa-nAChRα7 and Lsa-nAChRβ1as per their cluster group in the phylogenetic tree (Fig 1 and S2 Fig). Interestingly, one sequence was a divergent group member that clustered with *Drosophila melanogaster* β2, but with other nAChR α subunits from other arthropods. It was named Lsa-nAChRβ2 since it lacked the vicinal cysteines.

### Identification and characterisation of three genes encoding ancillary proteins in *L*. *salmonis*

An essential chaperone for nAChR expression in many species, including humans, is RIC-3 [24]. In addition to nAChR subunits, we identified this protein, and two other ancillary proteins (referred to as “APs”; RIC-3, UNC-50 and UNC-74). The open reading frame and lengths of the deduced protein for RIC-3, UNC-50 and UNC-74 are provided in the S2 Table. Multiple sequence alignment revealed that RIC-3, UNC-50 and UNC-74 in *L*. *salmonis* contained the respective conserved domains similar to these APs in other arthropods (S3 Fig).

### Functional expression of *L*. *salmonis* nAChR subunits along with three ancillary proteins

**Fig 2:**
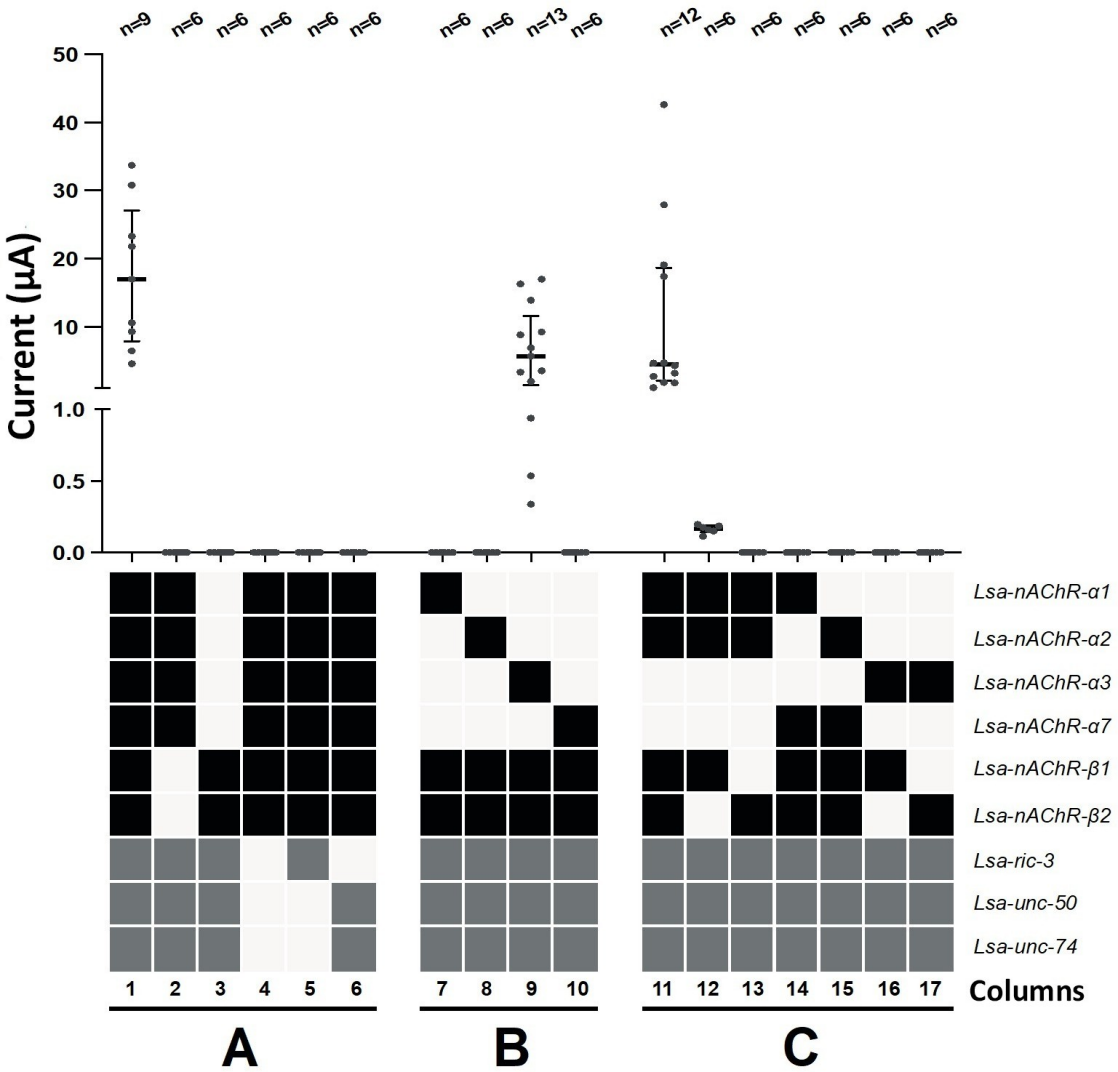
Determination of the minimum number of receptor subunits and ancillary proteins required for efficient expression of Lsa-nAChR. **A**) Co-injection (equimolar ratio) of six nAChR subunits (black squares) and the three ancillary proteins (grey squares; AP) yields strong currents with 100 μM ACh (1^st^ column). Injection of all alpha (α) or both beta (β) receptor subunits and APs failed to produce any measurable current (column 2 and 3). The same was true when APs were removed (column 4) or when Lsa-ric-3 or Lsa-unc-50 and Lsa-74 where combined with all the receptor subunits (column 5 and 6 respectively). **B**) Each individual nAChR-α subunit was co-injected with both nAChR-β subunit and APs (column 7 to 10). Only the Lsa-nAChR-α3 subunit was able to form a functional receptor gated by 100 μM ACh (column 9). **C**) The combination of Lsa-nAChR-α1 and Lsa-nAChR-α2 with both β receptor subunits and APs in oocytes challenged with 100 μM ACh also yields currents (column 11) while removing Lsa-nAChR-β2 (column 12) or Lsa-nAChR-β1 (column 13) from the injection mix almost or completely abolished the current. Combination of Lsa-nAChR-α1 or Lsa-nAChR-α2 with Lsa-nAChR-α7 with both β receptor subunits and APs (column 14 and 15 respectively) did not yield any current. Removing any of the β receptor subunit from the injection mix containing Lsa-nAChR-α3 (column 16 and 17) abolished the current in both cases. Grey points represent individual oocyte recording for each condition, black horizontal bar the median as well as the interquartile range. Numbers above the plot indicate the number of oocytes recorded for each condition. Y-axis is discontinuous to make the smaller currents recorded from the column 12 visible.

To determine the subunit composition of the functional *L*. *salmonis* nAChR channel, we tried different possible combinations of the six nAChR subunit (α1, α2, α3, α7, β1 and β2) cRNAs along with the cRNAs from the three AP, injected into *X*. *laevis* oocytes, which is one of the most commonly used systems for functional expression of nAChRs [5]. Three to five days after injection, the presence of a functional nAChR was investigated by the two-electrode voltage-clamp method. To determine the relative contribution of each gene for the functional expression of nAChR, we removed cRNAs from the injection mixture, either singularly or in combination (Fig 2). Co-injection (equimolar ratio) of the six nAChR subunits (black squares) and the three APs (grey squares) yields strong currents (17.00 μA [6.50 μA to 30.80 μA, median and 95% CI] with 100 μM ACh; Fig 2A, 1^st^ column). Injection of all alpha (α) or both beta (β) receptor subunits and APs failed to produce any measurable current (Fig 2A, column 2 and 3). The same was true when APs were removed (Fig 2A, column 4) or when only *Lsa-ric-3* or both *Lsa-unc-50* and *Lsa-unc-74* where combined with all the receptor subunits (Fig 2A, column 5 and 6 respectively). This highlights the crucial role of these ancillary proteins for expression of functional *L*. *salmonis* nAChR in *X*. *laevis* oocytes. Each individual nAChR-α subunit was co-injected with both nAChR-β subunits and APs (Fig 2B, column 7 to 10). Only the Lsa-nAChR-α3 subunit was able to form a functional receptor (5.72 μA [0.94 μA to 13.90 μA, median and 95% CI] with 100 μM ACh; Fig 2B, column 9). The combination of Lsa-nAChR-α1 and Lsa-nAChR-α2 with both nAChR-β subunits and APs also yielded currents (4.50 μA [1.88 μA to 19.10 μA, median and 95% CI] with 100 μM ACh; Fig 2C, column 11) while removing Lsa-nAChR-β2 (Fig 2C, column 12) or Lsa-nAChR-β1 (Fig 2C, column 13) from the injection mix almost or completely abolished the current (0.17 μA [0.11 μA to 0.20 μA, median and 95% CI] with 100 μM ACh and no current respectively). Combination of Lsa-nAChR-α1 or Lsa-nAChR-α2 with Lsa-nAChR-α7 with both β receptor subunits and APs (Fig 2C, column 14 and 15 respectively) did not yield any current. Removing any of the nAChR-β subunit from the injection mix containing Lsa-nAChR-α3 (Fig 2C, column 16 and 17) abolished the current in both cases.

**Fig 3:**
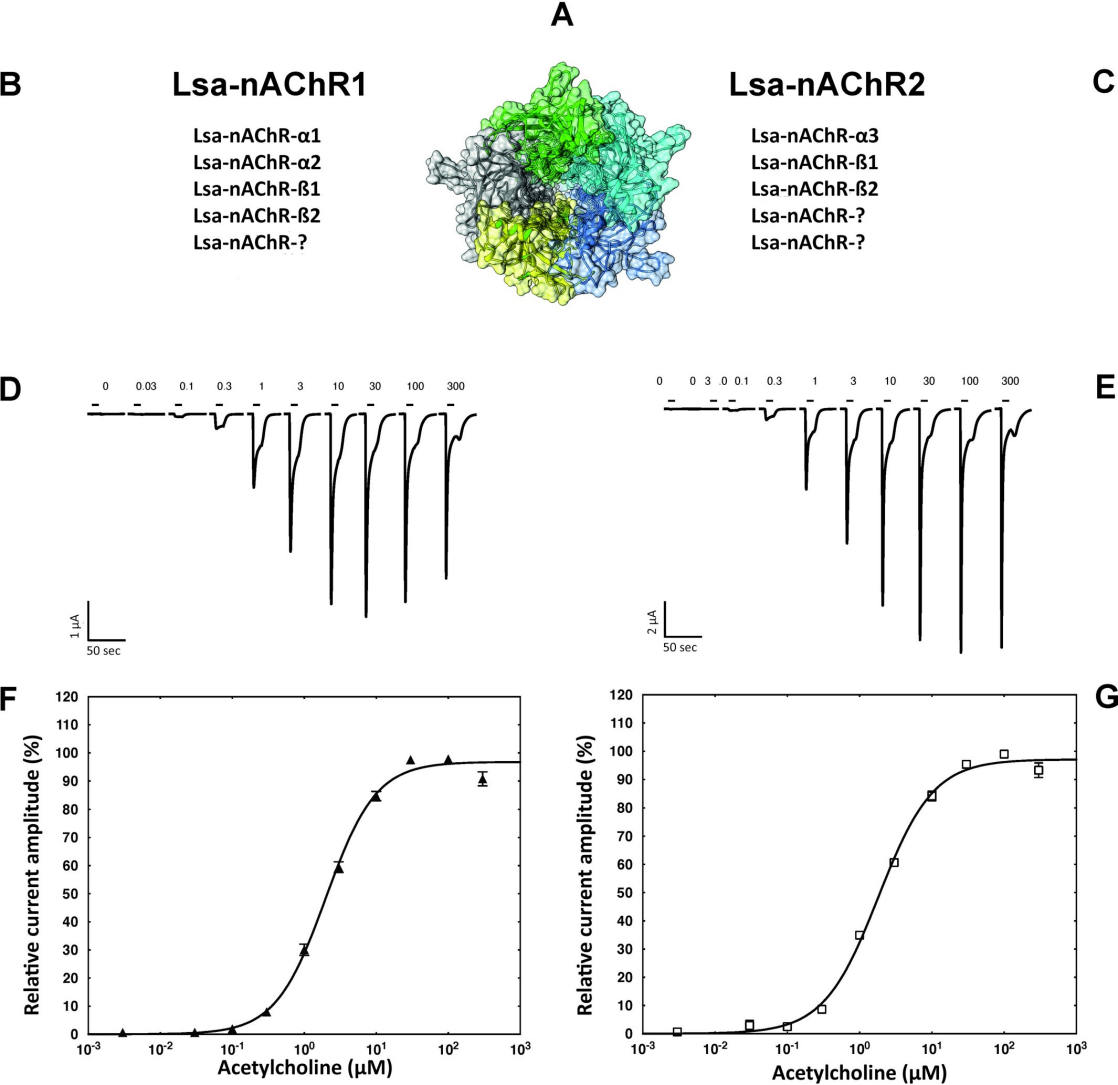
Hypothetical stoichiometry, current traces and dose response curves of *L*. *salmonis* nAChRs. Hypothetical 3D model of a Lsa-nAChR **(A**). Subunits composition as well as current traces and averaged dose response curves from a cumulative exposure to increasing dosage of acetylcholine (in μM) for Lsa-nAChR-1 (**B, D** and **F**) and Lsa-nAChR-2 (**C, E** and **G**) are shown. Individual curves were standardized to the fitted maximal current amplitude and subsequently averaged. Mean ± SEM of experiments carried out with at least six oocytes from two batches is shown. For both receptors, the exact stoichiometry is not known yet and the precise relative positioning of each subunit remains to be determined. The question marks (**B** and **C**) represent copies of one of the subunits already indicated. At high ACh concentration (300 μM; **D** and **E**), an open channel block is visible (smaller currents than at 100 μM) as well as bounce back current when the agonist is removed (relief from the agonist block).

The first receptor consisting of α1, α2, β1 and β2 subunits was named Lsa-nAChR1 while the second one made of α3, β1 and β2 subunits was named Lsa-nAChR2 (Fig 3B and 3C). Since these nAChRs are heteromeric pentamers, the fifth position in the assembly of Lsa-nAChR1 and the fourth and fifth positions in the assembly of Lsa-nAChR2 remains unknown (Fig 3B and 3C). A hypothetical 3D representation of an Lsa-nAChR1 consisting of one of each of the subunits α1, β1 and β2, and two of the subunit α2 was assembled *in-silico* using the Swiss model (https://swissmodel.expasy.org/) with the X-ray structure of the human α4β2 nicotinic receptor as template (https://www.rcsb.org/structure/5kxi) (Fig 3A). Typical current traces obtained from Lsa-nAChR1 or Lsa-nAChR2 are presented in the Fig 3D and 3E respectively. In both cases, the traces were characterized by a fast channel opening with almost no desensitization at low concentrations and a desensitization phase at higher concentrations (> 30 μM) with a steady state current. At a high ACh concentration (300 μM), an open channel block is visible (currents are smaller than at 100 μM) as well as bounce back current when the agonist is removed (relief from the agonist block). The calculated EC_50_ for ACh were 1.90 μM (1.62 μM to 2.37 μM, 95% CI, n = 12) and 1.83 μM (1.52 μM to 2.19 μM, 95% CI, n = 13) for Lsa-nAChR1 and Lsa-nAChR2, respectively (Fig 3F and 3G). Currents recorded from *X*. *laevis* oocytes expressing Lsa-nAChR1 or Lsa-nAChR2 and exposed to acetylcholine (Fig 3D and 3E) were significantly stronger (averaged maximum response >5 μA) than the ones from insect hybrid channels at identical acetylcholine concentrations as reported in the literature, e.g. by Bao *et al*. who recorded a maximum response to acetylcholine of a hybrid *Locusta migratoria*/*Rattus norvegicus* nAChR of 0.24 μA [25].

### Composition of Lsa-nAChR1 and Lsa-nAChR2

**Fig 4:**
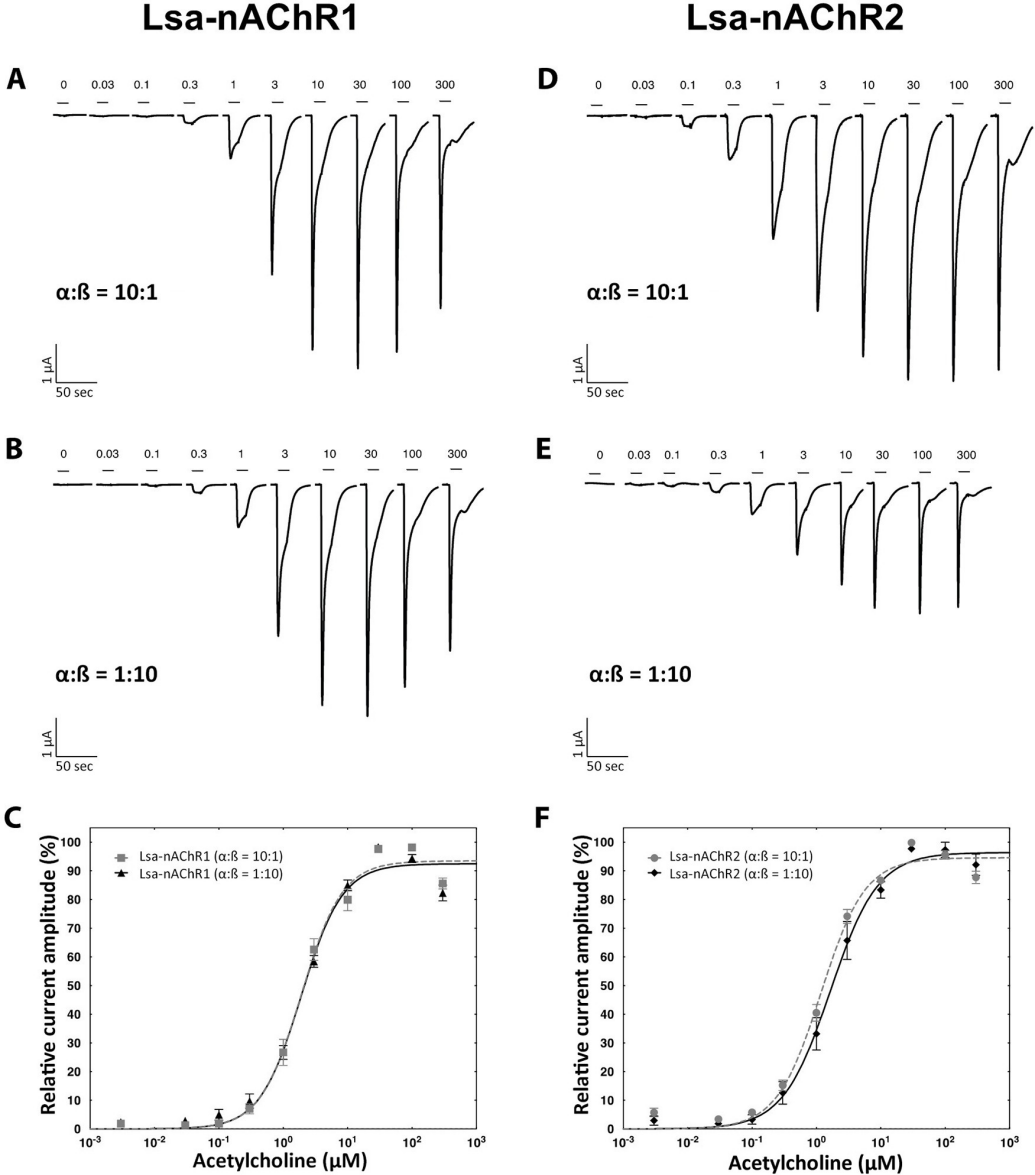
Effect of two different α:β ratio on Lsa-nAChR1 and Lsa-nAChR2 sensitivity to acetylcholine. Typical current traces and averaged dose response curves from a cumulative exposure to increasing dosage of ACh (in μM) for Lsa-nAChR-1 (A, B and C) and Lsa-nAChR-2 (D, E and F). The α:β ratio used for each experiment is indicated below the traces. Individual curves were standardized to the fitted maximal current amplitude and subsequently averaged. Mean ± SEM of experiments carried out with at least two oocytes from two batches is shown.

To identify if the two *L*. *salmonis* nAChRs could form different receptor subtypes, two different α:β equimolar ratios (α:β = 10:1; α:β = 1:10) were tested by biasing the amount of cRNA of both subunits in the injection mix. The effect of these ratios and in turn the different compositions of the ACh EC_50_ was determined. An EC_50_ value was generated for each individual cell and the mean EC_50_ and Hill Slope values subsequently averaged. As shown in the Table 1 and Fig 4 for Lsa-nAChR1, the different ratios tested had no statistically significant effect on the ACh EC_50_ values (mean EC_50_ at 2.11 μM ± 0.77 and 2.01 μM ± 0.71, respectively) (unpaired, two-tailed t-test: t = 0.308, df = 20, p = 0.761). For Lsa-nAChR2 (Table 1; Fig 4), the ACh EC_50_ values were significantly different between the ratio α:β = 10:1 (mean EC_50_ at 1.23 μM ± 0.37) and the ratio α:β = 1:10 (mean EC_50_ at 1.93 μM ± 0.69) (unpaired, two-tailed t-test: t = 2.43, df = 11, p = 0.033).

**Table 1:** Characterization of two different compositions of Lsa-nAChR1 and Lsa-nAChR2. The different combinations tested had no significant effect on the ACh EC_50_ value on Lsa-nAChR1 while a statistically significant lower ACh EC_50_ value was recorded with the α/β = 10:1 ratio for Lsa-nAChR2. "n = " refers to the number of individual cells evaluated.

Receptor ID	Lsa-nAChR1		Lsa-nAChR2	
Ratio (α/β)	10:1	1:10	10:1	1:10
Mean EC_50_ (μM) ± SD	**2.11** ± 0.77	**2.01** ± 0.71	**1.23** ± 0.37	**1.93** ± 0.69
Mean Hill Slope ± SD	1.46 ± 0.37	1.44 ± 0.52	1.30 ± 0.22	1.25 ± 0.23
Mean max current (μA)	4.85	8.35	2.52	3.17
n =	8	14	9	4

**Fig 5:**
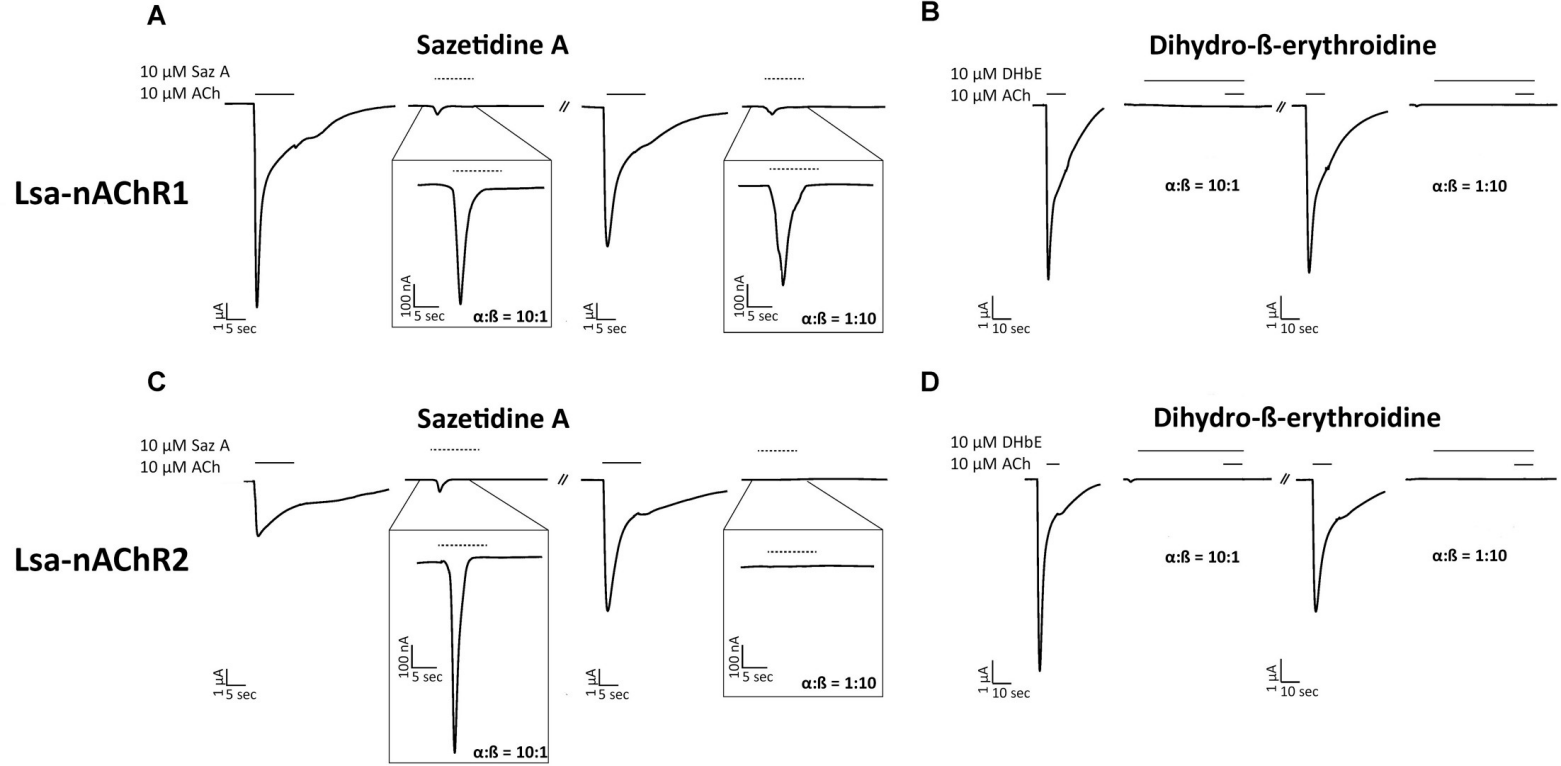
Effect of two different α:β ratio on Lsa-nAChR1 and Lsa-nAChR2 sensitivity to sazetidin A (Saz A) and dihydro-β-erythroidine (DHbE). Typical current traces obtained with 10 μM Saz A (A and C) or 10 μM DHbE (B and D) obtained from oocytes expressing two different ratios of α:β Lsa-nAChR1 (A and B) or Lsa-nAChR2 (C and D) are shown. The bars indicate the time period of ACh (black line), Saz A (interrupted line), or DHbE (grey line). The traces obtained with Saz A are also represented with a smaller scale to allow a better visualization of the peak currents recorded.

This indicates that Lsa-nAChR2 might form two different receptors subtypes with different compositions and affinity for ACh. Sazetidine A is a drug which acts as a subtype selective partial agonist of the vertebrate α4β2 neural nAChR, acting as an agonist at (α4)_2_(β2)_3_ pentamers, but as an antagonist at (α4)_3_(β2)_2_ pentamers [26]. *Xenopus* oocytes injected with two different ratios of α and β subunits (α:β = 10:1; α:β = 1:10) of Lsa-nAChR1 and Lsa-nAChR2 were exposed to 10 μM of sazetidine A (n = 3) after verifying a significant current evoked by a brief acetylcholine pulse. An inward current of, 0.24 μA, 0.22 μA, and 0.50 μA on average, respectively, was observed for both α and β subunit ratios of Lsa-nAChR1. In contrast, only one ratio (α:β = 10:1) of Lsa-nAChR2 was responsive to sazetidine A, the other was not (Fig 5A and 5C). This indicates that the Lsa-nAChR2 subunits α3, β1, and β2 are able to form two different receptor subtypes with different pharmacological properties, whereas Lsa-nAChR1 could not. Functional heteromeric AChRs, where the structure has been solved, contain at least two alpha subunits (reviewed in Millar 2003 [27]). Both β1 and β2 are required in both Lsa-nAChR1 and Lsa-nAChR2 (Fig 2B and 2C), which leaves only one subunit unaccounted for in the pentamers for both receptors. Studies to resolve this are ongoing. Dihydro-β-erythroidine (DHbE), a competitive nAChR antagonist with moderate selectivity for the neuronal α4 receptor subunit [28], was able to completely inhibit the ACh-triggered (10 μM) response in both Lsa-nAChR1 and Lsa-nAChR2 for both ratios tested when co-applied at 10 μM (Fig 5B and 5D).

### Pharmacology of *L*. *salmonis* nAChRs

**Fig 6:**
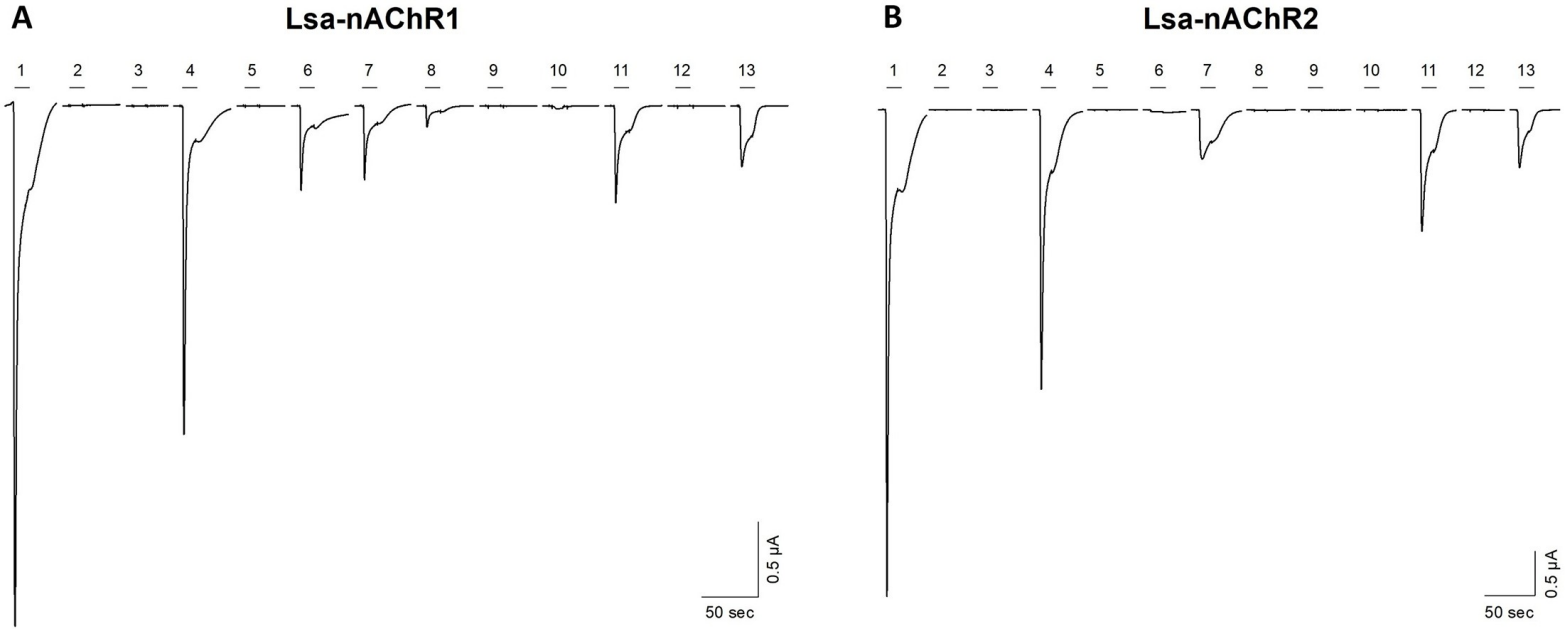
Agonist screening on Lsa-nAChR1 and Lsa-nAChR2. Typical current traces obtained with different agonists tested on Lsa-nAChR1 (A) or Lsa-nAChR2 (B). ACh (1) was tested at 100 μM) while all other compounds (2 to 13) were tested at 1 μM. 1) acetylcholine 100 μM; 2) azamethiphos; 3) emamectin; 4) nitenpyram; 5) deltamethrine; 6) thiacloprid; 7) clothianidin; 8) imidacloprid; 9) thiamethoxam; 10) acetamiprid; 11) dinotefuran; 12) cypermethrin; 13) acetylcholine 1 μM.

The pharmacological profile of the functional *L*. *salmonis* nAChRs in *X*. *laevis* oocytes was characterized by using cholinergic agonists. In addition, the effect of other drugs used as antiparasitic agents were tested to determine their affinity on nAChRs. Seven neonicotinoid compounds were tested to compare the relative sensitivities of the two Lsa-nAChRs (Lsa-nAChR1 and Lsa-nAChR2) to neonicotinoids. Besides, acetylcholine, nicotine and four non-nicotinoid compounds (azamethiphos, cypermethrin, deltamethrin, emamectin benzoate) were tested to determine their effect on these two Lsa-nAChRs. All the compounds were tested on *Xenopus laevis* oocytes expressing these Lsa-nAChRs injected at a 1:1 (α:β) ratio. In a first step, all compounds were tested at 1 μM using a pre- and co-application protocol (Fig 6). The advantage of this experimental protocol is the possibility to reveal agonistic effects during the pre-application and modulatory or inhibitory effects of the compound during the co-application. When tested on Lsa-nAChR1 (Fig 6A), nitenpyram, thiacloprid, clothianidin, imidacloprid, acetamiprid and dinotefuran were identified as agonists, while only nitenpyram, thiacloprid, clothianidin, and dinotefuran were so on Lsa-nAChR2 (Fig 6B).

As shown in Table 2, both ACh and nicotine act as agonist and partial-agonist, respectively, and could activate both Lsa-nAChR1 and Lsa-nAChR2. The calculated EC_50_ for nicotine were 3.95 μM and 9.98 μM with maximal peak currents compared to ACh of 27.9% and 16.1% for Lsa-nAChR1 and Lsa-nAChR2, respectively (Table 2 and S4 Fig). Only six of the seven nicotinoids were able to activate Lsa-nAChR1, whereas only three worked as agonists for Lsa-nAChR2. Thiacloprid was the most effective compound and worked as a partial-agonist of Lsa-nAChR1, with an EC_50_ measured at 12 nM only and a maximal peak current of 6.1% (compared to ACh; Table 2 and S4 Fig). Interestingly, it did not show any effect when tested on Lsa-nAChR2. The same was observed with imidacloprid but with a slightly higher EC_50_ (69 nM) and higher maximal currents (31.8%) on Lsa-nAChR1 and no effect on Lsa-nAChR2 (Table 2 and S4 Fig). Clothianidin, nitenpyram and dinotefuran all had EC_50_ measured in the low nanomolar range on both receptors and worked as partial-agonists, with the exception of dinotefuran that at high concentrations was a super-agonist of Lsa-nAChR2 (EC_50_ of 757 nM; Table 2 and S4 Fig). Acetamiprid was less effective with an EC_50_ at 10315 nM and worked as a partial-agonist of Lsa-nAChR1 only (Table 2 and S4 Fig). None of the non-nicotinoid compounds had any effect on Lsa-nAChR1 or Lsa-nAChR2 (Table 2 and S4 Fig).

**Table 2:** Comparison of sensitivities of Lsa-nAChR1 and Lsa-nAChR2 expressed in *Xenopus* oocytes to neonicotinoids and non-nicotinoid compounds. The compounds were tested on *Xenopus laevis* oocytes expressing these Lsa-nAChRs injected at a 1:1 (α:β) ratio. The table also gives results from an *in-vivo* assay in which salmon lice were exposed to seven neonicotinoids at 1 mg/L for 24 h. "n = " refers to the number of individual cells evaluated.

Drug	Lsa-nAChR1 (α1α2β1β2)				Lsa-nAChR2 (α3β1β2)				*L*. *salmonis* (preadult 2)
	EC_50_ (nM)	95% CI (nM)	Max peak (% Ach)	n =	EC_50_ (nM)	95% CI (nM)	Max peak (% Ach)	n =	Efficacy at 1.0 mg/L (%)
**Acetylcholine**	1,959	1,617 to 2,373	100	12	1,825	1,520 to 2,190	100	13	-
**Nicotine**	3,946	2,068 to 7,526	27.9	5	9,981	4,791 to 20,794	16.1	4	-
**Acetamiprid**	10,315	5,207 to 20,435	34.1	5	No effect	-	-	7	89.5
**Clothianidin**	37	23 to 61	41.9	7	81	61 to 108	75.4	4	54.3
**Dinotefuran**	220	155 to 313	69.8	7	757	630 to 910	110.2	6	100.0
**Imidacloprid**	69	36 to 134	31.8	8	No effect	-	-	6	100.0
**Nitenpyram**	45	24 to 85	27.4	8	381	317 to 459	70.1	4	17.7
**Thiacloprid**	12	5.5 to 26	6.1	5	No effect	-	-	6	100.0
**Thiamethoxam**	No effect	-	-	3	No effect	-	-	3	0.0
**Cypermethrin**	No effect	-	-	3	No effect	-	-	3	-
**Deltamethrin**	No effect	-	-	3	No effect	-	-	3	-
**Azamethiphos**	No effect	-	-	3	No effect	-	-	3	-
**Emamectin**	No effect	-	-	3	No effect	-	-	3	-

Further, we carried out *in-vivo* exposure of preadult salmon lice to all the seven neonicotinoid compounds at 1.0 mg/L (Table 2). Interestingly, three of the compounds (acetamiprid, imidacloprid and thiacloprid) did not affect Lsa-nAChR2, but still had a significant lethal effect on preadult parasites at this concentration.

## Discussion

The present study demonstrated the reconstitution, for the first time, of functional non-hybrid arthropod nAChR channels made of both species-correct α and β subunits in *X*. *laevis* oocytes. Co-expression of ancillary proteins, RIC-3, UNC-50 and UNC-74 was essential to obtain functional channels. These channels provide a better understanding of the native nAChR in an arthropod (*L*. *salmonis*) and could serve as a screening tool to provide the pharmacological characteristics of these native nAChRs. We identified eight different cDNAs encoding nAChR subunits (six α and two β) in *L*. *salmonis* (https://licebase.org/). Based on the presence of two adjacent cysteine residues, four of the subunits were classified as α subunits (α1- α3 and α7) and the remaining two were classified as β (β1 and β2) subunits.

Phylogenetic analysis revealed that all the six subunits in *L*. *salmonis* were closely related to nAChR subunits in different arthropods. The maximum relatedness was observed with the subunits from *Anopheles gambiae* and *Drosophila melanogaster* (Fig 1). All the α subunits clustered with the respective α subunits from other arthropods. However, one of the β subunits (β2) of *L*. *salmonis* grouped together with α8 subunits from other arthropods. Interestingly, nAChR β2 subunit from *Drosophila melanogaster* was also a part of the same cluster (Fig 1). The majority of arthropods possess only one β subunit, with a few exceptions such as *Drosophila melanogaster* [29], *Aedes aegypti* [30] and *Ixodes scapularis* [31]. Interestingly, only β1 in *Drosophila melanogaster* clusters with other β1 subunits in other arthropods, whereas β2 and β3 cluster with α subunits of other arthropods. A similar scenario has been observed in *L*. *salmonis*, with β1 subunits forming a cluster with β1 subunits from other arthropods and β2 subunits clustering with α8 subunits from other arthropods (Fig 1). The loss of the double cysteine residue of loop C of β2 in *L*. *salmonis* and *Drosophila melanogaster* might have been driven by a species-unique evolutionary process [31]. The majority of the nAChR subunits, both in vertebrates and invertebrates appear to be α subunits. In vertebrates 17 nAChR subunits have so far been identified, of which 10 are α subunits (α1-α10) and the number of β subunits varies from 1 to 4 (β1- β4) [4]. Similar scenarios have been reported in arthropods, with the majority of subunits defined as α (10 α subunits in insects), with only 1–5 β subunits (β1- β5). Members of only one β group (β1s) appear to be true orthologs of each other in arthropod species, suggesting that a single β subunit is involved in the common functional channel in arthropods [31]. In vertebrates, different β subunits combine with various α subunits to form different nAChR channels. Among the four β subunits in vertebrates, β1 forms a heteropentameric form in neuromuscular junctions [5] and β2 and β4 subunits are expressed in neuronal tissues and form functional channels [5]. Although β3 in vertebrates does not form a functional channel by itself with α subunits, co-expression with β2 subunit results in the formation of unique functional channels with α subunits, especially with α6 [5]. These results suggest that more pharmacologically and physiologically diverse nAChR channels are possible in vertebrates than in arthropods.

We also demonstrate here that two heteromeric acetylcholine and nicotine sensitive *L*. *salmonis* nAChRs could be readily reconstituted in *X*. *laevis* oocytes (Fig 2). Lsa-nAChR1 was expressed by injecting two α (α1 and α2) and two β (β1 and β2) subunits, whereas Lsa-nAChR2 was expressed by injecting only one α (α3) and two β (β1 and β2) subunits, respectively (Fig 3). In addition, *L*. *salmonis* APs (RIC-3, UNC-50, UNC-74) were needed for the expression of both the Lsa-nAChRs. These proteins are essential for correct folding, assembly and trafficking of the receptors to the cell membrane but are not part of the receptor itself.

To date, no group has succeeded in reconstituting a fully functional nAChR made of α and β subunits in an arthropod without co-expressing vertebrate subunits [21]. However, functional nAChRs formed by α subunits from *Drosophila* have been obtained by co-expression of the ancillary protein RIC-3 [16], which is an essential chaperone in many species, including humans [24]. The expression of both Lsa-nAChRs was efficient and robust in our system and the ACh-elicited currents could be measured in all the *X*. *laevis* oocytes expressing either of the Lsa-nAChRs. The peak current for both receptor types, obtained after exposure to acetylcholine, were significantly stronger (averaged maximum response >5 μA) than the ones from insect/vertebrate hybrid channels at identical acetylcholine concentrations [25]. This enables detection of subtle effects of xenobiotics on these receptors, which can be difficult to detect using arthropod/vertebrate hybrid nAChRs. It remains to be assessed if the combinations of subunits that failed to elicit any measurable current upon ACh application are well expressed, assembled and trafficked to the cell surface or possibly activates in the presence of another agonist. In addition, other APs not yet identified, might be necessary and required for the proper expression of some of the subunit combinations that did not respond to ACh.

In the current study, two different combinations of Lsa-nAChR2 seemed to be formed, although further experiments are necessary to confirm this. This is in line with observations of vertebrate nAChRs. When *Xenopus* oocytes are injected with α and β nAChR at a 1:1 ratio, the subunits normally assemble into a mixture of subpopulation of receptors with different stoichiometries, but they could also assemble into only one of the receptor types. For example, the human α4β2 nAChR, the most abundant nAChR subtype in the brain, assemble into a mixture of high- and low-affinity functional receptors, giving rise to biphasic ACh concentration-response curves when injected into *Xenopus* oocytes [32, 33]. High- and low-affinity human α4β2 nAChRs differ significantly in their functional and pharmacological properties [32–34] and result from the assembly of α4 and β2 subunits into two distinct stoichiometric arrangements: (α4)_2_(β2)_3_ (high-affinity subtype) and (α4)_3_(β2)_2_ (low-affinity subtype) [34]. Moroni and Bermudez have examined the functional and pharmacological properties of high- and low-affinity human α4β2 receptors using two-electrode voltage clamp procedures on *Xenopus* oocytes transfected with high (1:10) or low (10:1) ratios of α4/β2 cDNAs, which yield high (1:10)- or low (10:1)- affinity receptors with monophasic ACh concentration- response curves [35]. Thus, our data for Lsa-nAChR2 is consistent with the formation of two distinct receptors.

The *L*. *salmonis* APs were critical for the expression of both Lsa-nAChRs in *X*. *laevis* oocytes. Injection of cRNAs from all Lsa-subunits and Lsa-*ric-3* did not result in functional receptors. Injection of cRNAs for all subunits together with Lsa-*unc-50* and Lsa-*unc-74* also failed, while injection of cRNAs from all Lsa-subunits together with Lsa-*ric-3*, Lsa-*unc-50* and Lsa-*unc-74* resulted in functional receptors (Fig 2). Injection of all subunits together with Lsa-*ric-3* and either Lsa-*unc-50* or Lsa-*unc-74* were not tested.

RIC-3 (also known as resistance to inhibitors of cholinesterase 3) is a small transmembrane protein that belongs to the conserved family of *ric-3* genes coding for RIC-3 like proteins. RIC-3 acts as a chaperone and its activity is required within the cells that express nAChRs and is likely to affect the processes of receptor folding or assembly within the endoplasmic reticulum [36]. RIC-3 was an essential chaperone for the assembly of α-subunits to functional nAChRs in *Drosophila* [16]. The orthologs of *ric-3* have been characterized in vertebrates and invertebrates. The co-expression of *ric-3* gene is required for the maturation of multiple nAChRs in *C*. *elegans* [37]. The human RIC-3 is able to enhance the expression of *C*. *elegans* nAChR in *X*. *laevis* oocytes, demonstrating interspecies functionality. Besides, this shows that RIC-3 is likely to regulate nAChR-mediated transmission throughout evolution [38]. The other two APs required for the strong and robust expression were UNC-74 and UNC-50. The gene *unc-74* encodes a thioredoxin closely related to the human TMX3 protein [39] and this protein is likely to be required for the proper folding of nAChR subunits, although its function has not been characterized in detail. The gene *unc-50* encodes a transmembrane protein mostly localized to the Golgi apparatus and has been suggested to play a specific role in the regulation of nAChR trafficking [40].

The APs are evolutionarily conserved among species. However, our results indicate that these proteins are expressed at insufficient levels in *X*. *laevis* oocytes and hence no functional Lsa-nAChR could be obtained in the absence of these proteins. Alternatively, species-specific APs are required for the efficient expression of nAChRs. This could be one of the reasons why none of the earlier studies in arthropods have succeeded in expressing native nAChR made of α and β subunits in *X*. *laevis* oocytes. Therefore, the requirement of such APs might be worth considering when recombinant receptor expression fails. The fact that certain *D*. *melanogaster* nAChR α-subunits can only be expressed in *X*. *laevis* oocytes when they are co-injected with vertebrate β subunits may indicate that vertebrate β subunits are needed to recruit vertebrate ancillary factors and promote receptor expression in the absence of *D*. *melanogaster*-specific APs [18, 41]. Moreover, species-specific APs have been shown as a requirement for the expression of nAChR in nematodes as well [42]. The co-expression of *ric-3*, *unc-74* and *unc-50* has also been shown to be absolutely required for the expression of *C*. *elegans* and *H*. *contortus* levamisole-sensitive AChRs [42, 43].

Based on the EC_50_ estimated from our dose-response experiments (Table 1; Figs 4 and 5), Lsa-nAChR2 might form two receptors with different combinations of the subunits, whereas Lsa-nAChR1 had just one functional combination of subunits. Both Lsa-nAChR1 and Lsa-nAChR2 were sensitive towards ACh and nicotine (Table 2 and S4 Fig). However, the pharmacological characterization of these two *L*. *salmonis* nAChRs with neonicotinoid and non-neonicotinoid compounds demonstrated that these two nAChRs exhibit both similarities and differences in their sensitivity profiles towards these compounds (Table 2 and S4 Fig). None of the four non-neonicotinoid compounds tested had any agonist effect on either of the two nAChRs (Table 2). However, there were pharmacological differences in their responses to neonicotinoids. Out of the seven tested neonicotinoids, six acted as agonists for Lsa-nAChR1 whereas only three had agonist effects on Lsa-nAChR2 (Table 2 and S4 Fig). Besides, there were large differences in the EC_50_ values for clothianidin, dinotefuran and nitenpyram that acted as agonists for both Lsa-nAChR1 and Lsa-nAChR2 (Table 2 and S4 Fig). These three neonicotinoids have been reported to bind with high affinity to arthropod nAChRs and with low affinity to vertebrate nAChRs [10, 11].

The *in-vivo* assays with live parasites (Table 2) demonstrated that all neonicotinoids except thiamethoxam caused different degrees of mortality in preadult parasites. All except thiamethoxam also affected Lsa-nAChR1. Thiamethoxam is a neonicotinoid precursor converted to clothianidin in insects and has little efficacy on nAChRs on its own [44]. This could explain the lack of efficacy on nAChRs in the *Xenopus* model. For all compounds, no obvious relationship between the mortality and the concentration giving the *ex-vivo* effects on the receptor could be established. The discrepancy may have been caused by differences in kinetic properties of the compound in the parasite versus application of the compound directly to the receptor. In the *Xenopus* electrophysiology platform, the compounds are applied to the native receptor while when applied *in-vivo*, the compounds needs to 1) penetrate into the parasite, 2) avoid detoxification mechanisms, 3) reach the receptor at a sufficient concentration and 4) to produce a visible phenotype (e.g. paralysis or death). Three neonicotinoids (acetamiprid, imidacloprid and thiacloprid) did not demonstrate any *ex-vivo* effect on Lsa-nAChR2, but still gave high mortality rates in parasites exposed to 1 mg/L for 24 hours (Table 2 and S4 Fig). This observation could be due to the compound not reaching the receptor at sufficient concentrations for kinetic reasons. It could also indicate that there are more nAChRs in the parasite, not yet identified. A third possibility is that Lsa-nAChR1 is the main neuronal receptor type, and Lsa-nAChR2 has other functions. Further experiments using e.g. RNA-sequencing, *in-situ* hybridization and immunofluorescence antibody test (IFAT) are warranted. This would provide further insight to the involvement of these receptors in the biological processes of the parasite.

### Conclusions

The present study demonstrated that fully functional, non-hybrid nAChRs made of both α and β subunits from an arthropod could be reconstituted in *Xenopus laevis* oocytes and was only possible with co-injection of genes encoding three chaperone proteins. Using this approach, functional nAChRs from other arthropod pests, ectoparasites, disease vectors and pollinating insects can most likely be expressed in the *Xenopus* model. These results will significantly improve the current ways of obtaining nAChRs from arthropods, with a switch from hybrid nAChRs to species-specific native nAChRs.

## Materials and methods

### Ethics statement

Cultivation of parasites on Atlantic salmon requires an animal research license for the study, issued by the competent authority, the Norwegian Food Safety Authority. The license number for the current study was FOTS 7231. When sampling parasites, the fish were anesthetized with 100 mg/L metacaine. Studies on parasites do not require an animal research license in Norway.

### Chemicals

Compounds were purchased from Sigma-Aldrich (Buchs, Switzerland) except for Sazetidine A dihydrochloride, NS9283 and dihydro-β-erythroidine hydrobromide that came from Tocris (Tocris bioscience, a Bio-Techne brand, Switzerland). The modulatory compounds were prepared as 20 mM stock solutions in dimethyl sulfoxide (DMSO) and were dissolved in oocyte Ringer’s OR2 medium (see below), resulting in a maximal final DMSO concentration of 0.5% when compounds were tested at 100 μM. ACh was obtained from Sigma-Aldrich and prepared as stock solution at 100 mM in ddH_2_O.

### Identification and cloning of nAChR subunits and ancillary proteins

To identify the putative nAChR subunits, we used a BLAST algorithm (BLASTp) with known nAChR sequences from different arthropods to screen the *L*. *salmonis* genome and transcriptome (https://licebase.org/). Candidate *L*. *salmonis* nAChR subunits and APs were identified based on their considerable sequence similarity with previously characterized nAChR subunits, particularly at the N-terminal ligand-binding domain and the four transmembrane regions. For the cloning of the sea lice nAChR subunits and AP, RNA extraction, cDNA synthesis and PCR amplification were performed using previously described protocols [45]. In short, total RNA was extracted from one entire sea louse parasite from which, 1 μg of total RNA (DNase-treated) was reverse-transcribed to cDNA using a (dT)30 primer and SuperScript III Reverse Transcriptase (Invitrogen, Carlsbad, CA, USA) and the First choice RLM-RACE Kit from Ambion (AM1700, Waltham, MA, USA). Gene-specific primers (S3 Table) were designed using the Primer3 software (available at http://www.bioinfo.ut.ee/primer3-0.4.0). A rapid amplification of cDNA-ends by PCR (RACE-PCR) was performed using internal reverse primers (S3 Table) combined with the 5′ RACE Outer and Inner Primer (from the Ambion kit) to obtain the 5′-untranslated region (UTR). Internal forward primers (S3 Table) combined with a poly(dT) primer were used for the 3′-UTR of the transcript. Start and stop codons were deduced from the 5′ and 3′-RACE product sequences. The gene-specific PCR to obtain the full-length of subunits α1, α2, α3, α7, β1 and β2 from *L*. *salmonis* cDNA was performed with a Phusion polymerase (New England Biolabs, Ipswich, MA, USA) and their corresponding primer pairs (S3 Table). The reaction conditions were: 98°C for 30 s; 32 cycles of (98°C for 10 s; 60° C for 20s; 72°C for 45s); 72°C for 10min. PCR products were analysed on 1% agarose gels, excised, gel-purified using a NucleoSpin kit (Macherey Nagel, Düren, Germany), and cloned into pJET1.2 using the Thermo Scientific CloneJET PCR kit (catalog N° K1231, Waltham, MA, USA). Plasmid DNA was purified using the QIAprep Spin Miniprep Kit (Qiagen, Valencia, CA, USA) and at least three clones of each construct were sequenced using the provided pJET1.2 forward and reverse primers at Microsynth (http://www.microsynth.ch). Sequence quality check and assembly was performed using Geneious v5.6.7 [46] and a nucleotide blast was made on-line (NCBI) against the nucleotide collection (nt). The selected inserts were subcloned into a pT7-TS transcription vector (that introduces *X*. *laevis* ß-globin untranslated cDNA to the 5′- and 3′-end of the gene) via the restriction sites inserted in the primers (S3 Table). Plasmid DNA was purified with an EndoFree Plasmid Purification kit (Qiagen).

### Phylogenetic analysis

A phylogenetic analysis was performed using the six Lsa-nAChR subunit sequences and sequences for different nAChR subunits in other arthropods (*D*. *melanogaster*, *A*. *mellifera*, and *A*. *aegypti*). Protein sequences were aligned with ClustalW (S2 Fig) with a gap open cost and a gap extend cost set at 10 and 0.1 respectively. The phylogenetic tree was constructed using a maximum likelihood approach (PhyML 3.0; substitution model: blosum62) and bootstrap with 1000 iterations [47].

### Expression of *L*. *salmonis* nAChRs in *Xenopus laevis* oocytes

Capped cRNAs were synthesized (T7 mMessage mMachine kit, Ambion, Austin, TX, USA) from the linearized vectors containing the different subunits according to the manufacturer’s protocol. cRNA samples were stored at -80°C until use. Oocytes were ordered from Ecocyte (Germany). Oocytes were microinjected using a Roboinject automatic injection system (Multi Channel Systems, Reutlingen, Germany) with 15–25 nl of cRNA solution (30–300 ng/μl per subunit) and then incubated at 18°C in sterile filtered Barth solution containing: NaCl (88 mM), KCl (1 mM), NaHCO_3_ (2.4 mM), HEPES (10 mM, pH 7.5), MgSO_4_·7H_2_O (0.82 mM), Ca(NO_3_)_2_·4H_2_O (0.33 mM), CaCl_2_·6H_2_O (0.41 mM), at pH 7.4, and supplemented with 20 μg/ml of kanamycin, 100 U/ml benzylpenicillin and 100 μg/ml streptomycin. Recordings were made 3–5 days post-cRNA injection.

### Two-electrode voltage-clamp measurements using the HiClamp

Oocytes were impaled with two electrodes filled with 3 M KCl, and their membrane potentials were maintained at -80 mV throughout the experiment. Currents evoked by ACh or drugs were recorded using an automated process equipped with standard two-electrode voltage-clamp configuration (HiClamp, MultiChannel Systems). The principle of this system differs from standard electrophysiology because, instead of applying the compound in the perfusion, the oocyte is moved into a well from a 96-well microtiter plate containing the desired solution. Data were captured at 100 Hz, filtered at 10 Hz, and analyzed using proprietary data acquisition and analysis software running under Matlab (Mathworks Inc., Natick, MA, USA). Additional analyses were performed in Excel (Microsoft, Redmond, WA, USA). Plots of the peak inward currents as a function of the logarithm of the agonist concentration yield classical concentration-activation curves were readily fitted by single Hill equations. Concentration-activation curves were fitted with the equation:

$$Y=\frac{100}{1+10^{H(logEC50-X)}}$$ (1)

where Y is the normalized response, logEC_50_ is the logarithm of the concentration of agonist eliciting half-maximal current amplitude, X is the log of dose or concentration, and H is the slope factor or Hill slope. Individual curves were standardized to the initial ACh (100 μM) application amplitude and subsequently averaged. EC_50_ values were determined from the mean of at least 3 or more cells. Oocytes were washed with oocyte Ringer’s OR2 medium (82.5 mM NaCl, 2.5 mM KCl, 5 mM HEPES, 1.8 mM CaCl_2_·2H_2_O, and 1.8 mM MgCl_2_·6H_2_O, pH 7.4) and experiments carried out at 18°C.

To compare the ACh mean EC_50_ values obtained with the different subunit ratios injected for Lsa-nAChR1 and Lsa-nAChR2 (see Table 1) unpaired, two-tailed t-tests were conducted using an online calculator (https://www.graphpad.com/quickcalcs/ttest1/).

### Drug applications

Dose-response curves were obtained by sequential applications for 10s of increasing concentrations of ACh or drugs to oocytes expressing Lsa-nAChR1 or Lsa-nAChR2. A total time of 90 s time was allowed between applications for the channel to recover from any desensitization. To test the antagonistic effect of DHbE (Fig 5) and for the screening protocol (Fig 6), cells that evoked a significant current with ACh were exposed to the test compounds at 10 μM respectively 1 μM using a pre-application of 45 s and co-application of 10 s with ACh (1 μM).

### Biological assays

The exposure of preadult 2 parasites were conducted as described in [48]. The parasites originated from a salmon louse strain sensitive to all tested compounds [49]. The cultivation of salmon lice was conducted at the NIVA Marine Research Station Solbergstrand in the Oslofjord. Laboratory reared Atlantic salmon of were kept in 1 m^3^ tanks supplied with running seawater, salinity 33–35 parts per thousand, temperature 7–9°C. Salmon lice egg strings were harvested from adult females and hatched according to [50]. The preadult salmon lice were harvested on the day of exposure. Fish were anaesthetized with 100 mg/L metacaine for 2 min. The parasites were gently removed from the fish with forceps, placed directly in glass containers filled with 1000 mL filtered seawater and exposed to the compounds within 4 h.

Each group of parasites was exposed to 1 mg/L of the test substance for 24 h. The compounds were dissolved in DMSO and an emulsifier was added immediately before dilution with water. The bottles were finally incubated at 10°C according to [49], with continuous air supply, for 24 h.

After the incubation period was over, the solution with parasites was poured out through a funnel containing a filter. Parasites remaining attached to the bottle wall were classified as alive. Immobilized parasites were collected from the filter. These parasites were given a second chance to prove their swimming and attachment abilities in a Petri dish filled with fresh seawater. All parasites in the two groups were counted and classified according to sex and developmental stage.

## Supporting information

S1 Data Assembly files (bam-format) for reads mapping to a transcriptome file of genes coding for nAChR subunits in *L*. *salmonis*, generated from the predicted *L*. *salmonis* transcriptome (ftp://ftp.ensemblgenomes.org/pub/metazoa/release-47/fasta/lepeophtheirus_salmonis/cds/). The transcriptome file was constructed from the transcripts EMLSAT00000009264 (α1), EMLSAT00000000084 (α2), EMLSAT00000001002 (α3), EMLSAT00000005313 (α4), EMLSAT00000007472 (α6), EMLSAT00000003136 (α7), EMLSAT00000007263 (β1), EMLSAT00000006919 (β2).
(ZIP)

S1 Fig Protein sequence alignments of *L*.*salmonis* nAChR subunits. *Drosophila melanogaster* α1 nAChR sequence (CAA30172) is included for comparison. N-terminal signal leader peptides are underlined. The loops implicated in ACh binding (LpA-F) as well as the four transmembrane regions (TM1-4) are indicated. The two cysteines forming the Cys loop and the vicinal cysteines characteristic of α subunits are highlighted. The RRR motif are marked by asterisks (*). The N-glycosylation sites are boxed.
(PDF)

S2 Fig Protein sequence alignments for *l*. *salmonis* subunits. Protein sequences used for generation of the phylogenetic tree (Fig 1) in ClustalW alignment format.
(ALN)

S3 Fig Protein sequence alignment of RIC-3, UNC-50 and UNC-74. S3a Fig: Protein sequence alignment of *L*. *salmonis* RIC-3 with other RIC-3 proteins: Protein sequences from, *Apis mellifera* (AJE70270.1), *L*. *salmonis*, *Drosophila melanogaster* (NP_611537.2), *Caenorhabditis elegans (*NP_501299.1), *Danio rerio* (XP_017212528.2), *Xenopus laevis* (NP_001159914.1), *Homo sapiens* (AAP92162.1) and *Mus musculus* (XP_017177791.1). The two transmembrane domains (single line) and the coiled-coil domain are unlined (double line). S3B Fig: Protein sequence alignment of *L*. *salmonis* UNC-50 with other UNC-50 proteins: Protein sequences from, *Brugia malayi* (CDP93422.1), *Caenorhabditis elegans (*CAA88132.2), *Haemonchus contortus (*CDJ98119.1), *L*. *salmonis*, *Danio rerio* (NP_956541.1), *Xenopus laevis* (NP_001088522.1), *Homo sapiens* (NP_001317283.1) and *Mus musculus* (NP_001343457.1). Five transmembrane domains are underlined. S3C Fig: Protein sequence alignment of *L*. *salmonis* UNC-74 with other UNC-74 proteins: Protein sequences from L. salmonis, *Caenorhabditis elegans* (NP_491361.1) and *Haemonchus contortus* (ADV92278.1) and *Oesophagostomum dentatum* (JAA65034.1). C-terminal transmembrane domain and the Thioredoxin domain are underlined. Only these sequences were available in Genbank. This protein is uncharacterized in majority of species unlike other UNC proteins.
(PDF)

S4 Fig Typical current traces obtained from Lsa-nAChR-1 or Lsa-nAChR-2 upon exposition to selected compounds. The first trace corresponds to an acetylcholine control pulse (100 μM) that serves as the maximal peak current. The different concentrations tested (in nM) are indicated above each individual trace.
(PDF)

S1 Table The nAChR subunits identified in the *L*.*salmonis* genome: Six were α subunits and two were β subunits. Counts for each of the subunits from an RNaseq study of 16 individual adult *L*. *salmonis* females are provided. The transcriptome file was generated from the predicted *L*. *salmonis* transcriptome (ftp://ftp.ensemblgenomes.org/pub/metazoa/release-47/fasta/lepeophtheirus_salmonis/cds/). Assembly files (bam-format) for reads mapping to the transcriptome file in the folder S1 Data.
(PDF)

S2 Table The putative open reading frame and protein length of *L*. *salmonis* nAChR subunits and ancillary proteins, along with their GenBank accession numbers. (PDF)

S3 Table Primers used for PCR amplification of nAChR subunits and ancillary proteins from *Lepeophtheirus salmonis*. (PDF)
