# Supplementary material for: Nicotinic acetylcholine receptors: Ex-vivo expression of functional, non-hybrid, heteropentameric receptors from a marine arthropod, Lepeophtheirus salmonis
Source: PLoS Pathog. 2020 Jul 27;16(7):e1008715. doi: 10.1371/journal.ppat.1008715 (PMC7419010; doi:10.1371/journal.ppat.1008715)
Supplement: S1 Fig — Drosophila melanogaster α1 nAChR sequence (CAA30172) is included for comparison. N-terminal signal leader peptides are underlined. The loops implicated in ACh binding (LpA-F) as well as the four transmembrane regions (TM1-4) are indicated. The two cysteines forming the Cys loop and the vicinal cysteines characteristic of α subunits are highlighted. The RRR motif are marked by asterisks (*). The N-glycosylation sites are boxed. (PDF) [file ppat.1008715.s002.pdf]

```
Ls-nAChRa7     -------MNVIMIIIVMVYFSPTVFGGQTEKKVLNFVFNNYNILERPVRDPKEPVQLSMG 53
Ls-nAChRb1     --MDWNIMLIILLTF-LLRLFSPGHGSQEEERLVRDLFRGYNKLIRPVQNMTQKVEVAFG 57
Ls-nAChRa2     -------MLFILIF--LSFFYNPSNGNPDAKRLYDDLLSNYNRLIRPVSNHTEKVTVKLG 51
Ls-nAChRa3     MDKVWRISL-------IFVMITGSCGNPDAKRLYDDLLSNYNKLVRPVVNVSDAVTVKLK 53
Ls-nAChRb2     ---MFLIIIYKVLFVIVVFTEHARGGNPHAKRLYDDLLSNYNRLIRPVSNNTEKLTVRLG 57
Ls-nAChRa1     -MDTWTHSLCVLFFIGT-VYFSSVCANPDAKRLYDDLLSNYDRLIRPVQNSSDRLTVKMG 58
D        a1    ----MGSVLFAAVFIAL-HFATGGLANPDAKRLYDDLLSNYNRLIRPVQNNSDRLTVKMG 55


--LpD--                                                             --LpA----
Ls-nAChRa7     VSLRRIIGLDEKNQILTANLWITLIWKDHNLHWNRSEYPKIERLQIPTEHVWTPDILLYN 113
Ls-nAChRb1     LAFIQLINVNEKNQIMKSNVWLRFVWNDYQLQWDEADYGGISVLRLPPDKVWKPDIVLFN 117
Ls-nAChRa2     LRLSQLVDLNLKDQILTTNVWLEHEWKDYKFTWDPSEYGGVTEIYVPSEHIWLPDIILYN 111
Ls-nAChRa3     LKLSQLIDVNLRNQIMTTNLWVEQFWYDYKMRWEPDEYGGVDMLHVPSDHIWRPDIVLYN 113
Ls-nAChRb2     LKLSQLIDVDLKNQVMTTNVWVEQKWLDYKLNWKPEEYGGVEMLYVPSQHIWLPDIVLFN 117
Ls-nAChRa1     LKLSQIIGIDMKRQIMITNVWVAQEWHDYKLKWKPEDYGGVKHLHVPSQNIWLPDIVLYN 118
D        a1    LRLSQLIDVNLKNQIMTTNVWVEQEWNDYKLKWNPDDYGGVDTLHVPSEHIWHPDIVLYN 115

                     -------LpE------          Cys-loop          --LpB-
Ls-nAChRa7     SASTAFNNFYHVNLLLSNEGEIRYMPPGIIQSTCEIDITWFPFDEQNCTFKFGSWTHDGN 173
Ls-nAChRb1     NADGNYEVRYKSNVLIYPDGEVLWVPPAIYQSSCTIDVTYFPFDQQTCIMKFGSWTFTGD 177
Ls-nAChRa2     NADGDYIVTTMTKAILHYDGKVVWTPPAIFKSSCEIDVEFFPFDKQTCFLKFGSWSFDGF 171
Ls-nAChRa3     NADGNFEVTLSTKATLFNTGLVVWRPPAIYHSSCEMDVEYFPFDEQTCVMKFGSWTYDGF 173
Ls-nAChRb2     NADGKYEVTLMTKARLKYTGEVVWKPPAIYKSSCKINVEWFPFDEQSCDMKFGSWTYDGF 177
Ls-nAChRa1     NADGNYEVTIMTKAVLHFSGRVVWNPPAIYKSSCEIDVEFFPFDEQKCFMKFGSWTYDGY 178
D        a1    NADGNYEVTIMTKAILHHTGKVVWKPPAIYKSFCEIDVEYFPFDEQTCFMKFGSWTYDGY 175

               -    ---LpF-------               -------LpC------------
Ls-nAChRa7     RLNLSMMDSKG---------DLTNFETSVEWEVLGMPAIRTEDIYDCCKD-------KIF 217
Ls-nAChRb1     QVSLTLYNNKD-------YVDLSDYWKSGTWDIIEVPAYLNIHNDTL----------PTE 220
Ls-nAChRa2     QVDLVHINSQPDNDTVSYGMDLSEYYLNVEWDILEVPAERHVKTYPCCP--------EPY 223
Ls-nAChRa3     QVDLRHQEEEQGTNVVNIGVDLSEFYMSVEWDILAVPAIRNVKVYTCCD--------EPY 225
Ls-nAChRb2     QVDLKHVDQEKGSNVVKVGIDLKEFYYSVEWDILEVPATRNEEYYTNDRGEEDDYQGKLL 237
Ls-nAChRa1     MVDLRHINQKGSSSEIEIGMDLQEYYISTEWDVMTAPAVRNEKYYPCCE--------EPY 230
D        a1    MVDLRHLKQTADSDNIEVGIDLQDYYISVEWDIMRVPAVRNEKFYSCCE--------EPY 227

               --      RRR           -------TM1--------     ------TM2--
Ls-nAChRa7     QDITYTIQLRRRTLYYLGNWTLPCVLIASMAILGFYFPPESGEKITLEITILMSLTFFMN 277
Ls-nAChRb1     TDISFYITIRRKTLFYTVNLILPTVLISFLCVLVFYLPAEAGEKVTLGISILLSLVVFLL 280
Ls-nAChRa2     PDIYFSIIIRRKPLFYVVNLIIPCVGIFYLSILVFYLPAQSGEKTALVIAILVSQTLYFT 283
Ls-nAChRa3     LDITFNITMRRKTLFYTVNLIIPCMGISFLTVLVFYLPSDSGEKVSLSISILLSLTVFFL 285
Ls-nAChRb2     TDITFMMTLRRKTLFYTVNLIIPCVGISFLTVLVFYLPSDSGEKVTLCISILLSLTVFFL 297
Ls-nAChRa1     PDIIFYLTLRRKSLFYTVNVIIPCVGISFLSVLVFYLPSDSGEKVSLSISILLSLTVFFL 290
D        a1    LDIVFNLTLRRKTLFYTVNLIIPCVGISFLSVLVFYLPSDSGEKISLCISILLSLTVFFL 287
                          *
               ------      ---------TM3-----------
Ls-nAChRa7     MVTDMQPPSS-KTPLVGIYFSCIMIMIASSVICSILVINYHHRLTEYGDMPKWFRFIFFK 336
Ls-nAChRb1     LVSKILPPTSLVLPLIAKYLLFTFIMNTVSILVTVVIINWNFRGPRTHSMPNWIRVLFLK 340
Ls-nAChRa2     LVIEVIPATSKTLPLLGRYLIFSMIFIAIAVTLTTIILNLHYRKPSTHRMPQWVRRTFIQ 343
Ls-nAChRa3     LLAEIIPPTSLVVPLLGKFVLFTMILDTFSICVTVVVLNVHFRSPQTHTMAPWVRRVFIH 345
Ls-nAChRb2     LLAEIIPPTSLAVPLLGKYLLFTMILVTLSICVTVGVLNIHFRSPATHRMSPWMRKIFIH 357
Ls-nAChRa1     LLAEIIPSTSIAVPLLGKYLLFTMILVTFSVMLTIGVLNVNFRTPATHKMAPWVRKTFVE 350
D        a1    LLAEIIPPTSLTVPLLGKYLLFTMMLVTLSVVVTIAVLNVNFRSPVTHRMAPWVQRLFIQ 347
```

```
Ls-nAChRa7     VLPKILLMSFPNQENLASGTASKKSMEM----------------EL-------------- 366
Ls-nAChRb1     YLPIVLFMRRPKKTRLRWMMEMPGQSRR-----------IPPP------HPSYCPPPTNN 383
D       a1     RLPRILLMRVPIQVIKDTMKTRRSK---------Y----------LR------------- 371
Ls-nAChRa3     ILPRLLVMRRPGQSPESKMRSRPMI--KENGYGHYMIEQVRLQHVIQQ------------ 391
Ls-nAChRb2     IMPRMLLMTRPHYIPRYSAEPPLKSRSND--------------------DL-------MY 390
Ls-nAChRa1     FLPRFLFIRRPEKEEEEEEEVLFTPETECDEKSQALLQSSQPPMFLQKEDLLKIPPPFSL 410
D       a1     ILPKLLCIERPKKEEPEEDQPPE---------------------VL--TDVYHLPPDVDK 384


Ls-nAChRa7     ----------------EDLHSDAF-----ESSS------LK------------------- 380
Ls-nAChRb1     GTSSSSNNNN---NTSNNNHQ---HHPHPDSSSSPLSGGIKLTAPNAPLPPPPPVSPPSS 437
Ls-nAChRa2     QSDPALKSLAGKYGEDDDEEDDAK---GNGGLNSQLRGHL-------------------- 408
Ls-nAChRa3     ---RESNHINVHYEASEDPETAY-ESKQNY-KNSHFHDNNN------------------- 427
Ls-nAChRb2     NQDQDSKKLNGHCGPTTN------------------------------------------ 408
Ls-nAChRa1     D--HDEKSLQR-----ISPSRDDIQSKISPSSGDPFYGSGNSGFPLY----PLPLGGIDS 459
D       a1     FVNYDSKRFSGDYGIPALPASHRF--DLAAAG-----GISAHCFAEPPLPSSLPLPGADD 437


Ls-nAChRa7     ------------------------------------------------------MEDLYS 386
Ls-nAChRb1     DLIKNKIEIMELNDMQNTNSSQPQPPNSSSHIHHPTCKLNQSHQGSFDDEEVEDEDDDLT 497
Ls-nAChRa2     -------------------------------------------NGLY-----RGLTKI-M 419
Ls-nAChRa3     -------------------------------------------------------TEIFD 432
Ls-nAChRb2     ------------------------------------------------------------ 408
Ls-nAChRa1     SKDS----VSEL------------------------------VPTFYGGSESMGYHGDYS 485
D       a1     DLFS---------------------------------------P--------SGLNGDIS 450


Ls-nAChRa7     PVP------------------DHCPVHKYNIQEIEEDENSPQITE-------------VS 415
Ls-nAChRb1     TSPTGRGNHHHPPPPHHHQHLHHHHHHHLDEEEDQAQQHPPPPHHRGGRRNSDEDDGSEA 557
Ls-nAChRa2     TST--------------------CDVM----DVKS------------------------S 431
Ls-nAChRa3     YSS--------------------CELH----GTPENLPSPPPPLNPSF------IYPGPN 462
Ls-nAChRb2     EDQ--------------------CKKN----GKDEQDS-----TKPN-----------KK 428
Ls-nAChRa1     --S--------------------CERH-----LDKA-----------------------H 495
D       a1     PGC--------------------CPAA----AAAAAAD-----LSPTF----------EK 471

                                                         --------TM4-------
Ls-nAChRa7     CLDATLHDILIEIQVITNKIEEQDFHSEKKNEWKLAAMVLDRFCLITFTLLTILLTAAVV 475
Ls-nAChRb1     FLSPEAYRATQAVEFIAEHLRNEDEYLQIREDWKFVAMVVDRMQLYVFFIVTTVGTVGIL 617
Ls-nAChRa2     TSPFAIEKAVHNIMFIKHHMKRQDEFDAEDQDWGIVAMVLDRLFLWVFGISALVGSIMIL 491
Ls-nAChRa3     GECPEVYRALDGVRYIAECTKREEDSCKVKEDWKYVAMVMDRLFLWIFTIAVLVGSAGII 522
Ls-nAChRb2     KSISNVACALRGVEFIAQHIKNADKDNEVIEDWKFIAMVMDRLFLWLFTIACIMGTGGII 488
Ls-nAChRa1     ELNPALEKAMNALKFVAQHVKNEDNFESFSDDWKYVAMVLDRILLWVFTVACVFGTAGII 555
D       a1     PYAREMEKTIEGSRFIAQHVKNKDKFESVEEDWKYVAMVLDRMFLWIFAIACVVGTALII 531

               -
Ls-nAChRa7     IASPQVIVW---------------------------------   484
Ls-nAChRb1     MDAPHIFEYVDQDKIIDIYKGK--------------------   639
Ls-nAChRa2     VESPNMYEEVSP---IDVIFSKIALEESSRVSQEKV--FM--   526
Ls-nAChRa3     LQAPALYDTRAA---IDVELSQIEAATAKPLSEQRNKFSFLK   561
Ls-nAChRb2     LRAPSLYDMRDP---IDAKLSEIPKF----------------   511
Ls-nAChRa1     MAAPSHQDTRQP---IDIQFSKVDKISA--LRMMPESLKSQR   592
D       a1     LQAPSLHDQSQP---IDILYSKIAKKKFELLKMGSENTL---   567
```

Fig S1: **Protein sequence alignments of *L.salmonis* nAChR subunits.** *Drosophila melanogaster* α1 nAChR sequence (CAA30172) is included for comparison. N-terminal signal leader peptides are underlined. The

loops implicated in ACh binding (LpA-F) as well as the four transmembrane regions (TM1-4) are indicated. The two cysteines forming the Cys loop and the vicinal cysteines characteristic of α subunits are highlighted. The RRR motif are marked by asterisks (*). The N-glycosylation sites are boxed.
