# Supplementary material for: Nicotinic acetylcholine receptors: Ex-vivo expression of functional, non-hybrid, heteropentameric receptors from a marine arthropod, Lepeophtheirus salmonis
Source: PLoS Pathog. 2020 Jul 27;16(7):e1008715. doi: 10.1371/journal.ppat.1008715 (PMC7419010; doi:10.1371/journal.ppat.1008715)
Supplement: S3 Fig — S3a Fig: Protein sequence alignment of L. salmonis RIC-3 with other RIC-3 proteins: Protein sequences from, Apis mellifera (AJE70270.1), L. salmonis, Drosophila melanogaster (NP_611537.2), Caenorhabditis elegans (NP_501299.1), Danio rerio (XP_017212528.2), Xenopus laevis (NP_001159914.1), Homo sapiens (AAP92162.1) and Mus musculus (XP_017177791.1). The two transmembrane domains (single line) and the coiled-coil domain are unlined (double line). S3B Fig: Protein sequence alignment of L. salmonis UNC-50 with other UNC-50 proteins: Protein sequences from, Brugia malayi (CDP93422.1), Caenorhabditis elegans (CAA88132.2), Haemonchus contortus (CDJ98119.1), L. salmonis, Danio rerio (NP_956541.1), Xenopus laevis (NP_001088522.1), Homo sapiens (NP_001317283.1) and Mus musculus (NP_001343457.1). Five transmembrane domains are underlined. S3C Fig: Protein sequence alignment of L. salmonis UNC-74 with other UNC-74 proteins: Protein sequences from L. salmonis, Caenorhabditis elegans (NP_491361.1) and Haemonchus contortus (ADV92278.1) and Oesophagostomum dentatum (JAA65034.1). C-terminal transmembrane domain and the Thioredoxin domain are underlined. Only these sequences were available in Genbank. This protein is uncharacterized in majority of species unlike other UNC proteins. (PDF) [file ppat.1008715.s004.pdf]

```
Apis            -------------------------------MAEITDFGPRKTIFILAIVAGCFAVLWPK    29
Lsa-RIC-3       ---------------------------MADKSFNMSDVSTGKSILICGIILRCFAVLWPK    33
Drosophila      -MPATA-------------------TSKPRAPLVEEGMTPKKTALIIVTVIGCIAILWPK    40
C elegans       -MPKTERRRDRDRDRDRERRNRRKRDDSYDDYDEEGGISGWKLGLVFGVIVVCFAMLYPT    59
zebrafish       MFSVISASSGQKRCRDVDVRHR-LLCGVFSLSAIDMAISTCQKITFVSCLVLCMSLFLPR    59
Xenopus         -----------------------------------MALSAVQKVVLFSCLVLCVSLLLPR    25
human           -----------------------------------MAYSTVQRVALASGLVLALSLLLPK    25
mouse           -----------------------------------MAYSTVQRVALASGLVLAVSLLLPK    25

Apis            IFYPMLTASV---NPHHIT------DNSACCGVIFESDVTAADIMYEICQNILKHHQIDP    80
Lsa-RIC-3       IFHPMLFGSN---LFSTKNA----------------------------------------    50
Drosophila      VFHPMMFGGV---PPSQPNFKDPRAAPGGCCDVVLDREQFLNAT---------K------    82
C elegans       LFHPMLMGFLGRSPPSSPSINQQRPPI---------------------------------    86
zebrafish       LFLPRVKKEA---VRS--DVGPGHLPPS--------------------------------    82
Xenopus         AYIARGKQAA---Q----EGNTGLFQS---------------------------------    45
human           AFLSRGKRQE---PPPTPEGKLGRFPPM--------------------------------    50
mouse           AFLSRGKRPE---PPPGPEGKLDRFPPM--------------------------------    50

Apis            RIRDALRTIKLTPQSASLCREEILARCGIDLSTFLAKREHLEKSYKQVLEEIRSFNSSLC    140
Lsa-RIC-3       ------------------------------------------------------------    50
Drosophila      ---------------------------------------------KDT---VEPF-----    89
C elegans       ------------------------------------------------------------    86
zebrafish       ------------------------------------------------------------    82
Xenopus         ------------------------------------------------------------    45
human           ------------------------------------------------------------    50
mouse           ------------------------------------------------------------    50

Apis            LKINFGIPLSQLGTPHLIRY--HILM-PHNTIKQERRTPPHAGGLHPALRERGRAIPSSH    197
Lsa-RIC-3       ---------------------------VKDSLRQERSSDMHPEYAHPAFRERGKIFYGEE    83
Drosophila      -------------GPHLYRKQINVYT-GEISLRQERPAHLHPESIYQAMRERGRAIPATP    135
C elegans       ----------------------HPAMGGGSG----QRHPGGGADVHPAMRMA--------    112
zebrafish       --------------R-------HIHSLSEDH----DHWDTDSHYIKHYSPDA--------    109
Xenopus         --------------P-------GQNSKPTDG------RPGGAHFPRSHMAEA--------    70
human           --------------M-------HHHQAPSDG------QTPGARFQRSHLAEA--------    75
mouse           --------------M-------HHHSAPSDG------QTPGARFQRSHLAEA--------    75

Apis            IVPKV-------------SDRPDHVVPKMRPPLGGAGHVVPAPKGSGTMGIIMPLYTLGI    244
Lsa-RIC-3       GRQIK-------RSMDKDMRAGPGPVPGMRPTFGGPGMPTPAPRSQGTMGVLMPMYTIGI    136
Drosophila      TVPILERKTSPSNPPPRIVDGRPGPIPGMRPPMGAGALHQPQQR-GSSMGFLMPLYTIGI    194
C elegans       ----------------------------QAQAES------QSGGSKGMFTWMLPVYTIGV    138
zebrafish       ----------------------------IAS--------FKGVKKPNLLGQVIPVYGFGI    133
Xenopus         ----------------------------VSKAKGGTGG-GGGGTRPSLVGQIIPIYGFGI    101
human           ----------------------------FAKAKGSGGGAGGGGSGRGLMGQIIPIYGFGI    107
mouse           ----------------------------FAKAKGAGGGAGGGGSGRGLMGQIIPIYGFGI    107

Apis            VLFFLYTIVKVLRKNSDSEIISEYPGAAAEK------EFRKMVFSPEA------------    286
Lsa-RIC-3       IVFFVYTTMKIIFKKNDDP----IN----EP------LYSRKSLNSGMEEIQDVSESPTR    182
Drosophila      VVFFGYTLMKIMFKKQVPND--PYGAAPPNP------AFRQEVFGSQN------------    234
C elegans       VLFLLYTLFKSKGKKSKRKKRNYFDSEDDDDESESETKYGGKFGKKKLEGLQK-------    191
zebrafish       FLYIIYLFVKLTSKDKPHRQGCRFPLLQS-------QYTFEEMPTCQLVQLQA-------    179
Xenopus         LLYILYILFKLSSKGKNAKLEISTQPVAN-------GNLKRKINDYELIQLQD-------    147
human           FLYILYILFKLSKGKTT-AEDGKCYTAMP-------GNTHRKITSFELAQLQE-------    152
mouse           FLYILYILFKLSKGKT--AEDRNCSTAPP-------GNAHRKITNFELVQLQE-------    151

Apis            FA----TAMTG---------------------GTMNYQKER--SPSPQR-----------    308
Lsa-RIC-3       YPGAAVDDYCGNSLYITKKINENVETLHHEILGNEHYQQQKQVTPTGIPEDHSISRCNDA    242
Drosophila      --HSQVEDLGGSKL------------------GAATATAAA-KKPAAKDT----------    263
C elegans       ------------------RLRETES--------AMSKILEQLESVQAGAN----------    215
zebrafish       ------------------RMSEK----------AREKRISKVVHPSARSR----------    201
Xenopus         ------------------KLKETEE--------AMEKIISR-LGPNCESA----------    170
human           ------------------KLKETEA--------AMEKLINR-VGPNGESRA---------    176
mouse           ------------------KLKETEE--------AMEKLINR-VGPNGE-RA---------    174

Apis            --------------------------------PTPTLEELKDLDG---------------    321
```

```
Lsa-RIC-3      ACRQINNAKIMTDRTEN----LLIGNNKVMDTGDPRDVEINLLRQRL-EETEKAMERIMA    297
Drosophila     -EKELYNASVSATEVASSLSASLKSHQQL--KEAEQLMEIEKLRQKL-ESTERAMAQLVA    319
C elegans      --------PVDLDAADKRSEQLE--------EDPSVKEAV----GLT----ETNEQYIK    250
zebrafish      ---------RGTRRREERKLKQLK--------EISQMMRERQLREGATPEEEAEEAPYNA    244
Xenopus        --------DNMSSDEEIHLLQRLK--------EITRVMKEGKTLDGISPEKEAEEAPYME    214
human          --------QTVTSDQEKRLLHQLR--------EITRVMKEGKFIDRFSPEKEAEEAPYME    220
mouse          --------QAVTSDQEKRLLHQLR--------EITRVMKEGKFIDT-SPEKEAEEAPYME    217

Apis           -------PPKS------------KGLAG-IDRSSK---DNT---------IAQVQYSTED    349
Lsa-RIC-3      QMGGLVQNPKNESEVVEHLPEEESKEEG-LEKNNC---DDKSGSISEQETDGE------E    347
Drosophila     EMNTDQYEAKKN-------DNEKTREQP-LDKQNL---SNGHASSTDQNPEQETVAKGAR    368
C elegans      DLEVAL--------------KEFQSLSKEYDKAKMKKLKRKDSSSDEDEED---------    287
zebrafish      DWEGYP--------------DETY---PEYKIPSR---RRRYPSVILEEPDQV-------    277
Xenopus        DWEGYP--------------EETY---PVYDPSDY---KRTQQTILVDCSLLN-------    247
human          DWEGYP--------------EETY---PIYDLSDC--IKRRQETILVDYPDPK-------    254
mouse          DWEGKM--------------PLPC------------------------------------    227

Apis           KVENIEHSPTIKVMGMEMTASCENKGSR---PTTPIIPI--SPSHIEREKTPPKPIYLEG    404
Lsa-RIC-3      SR-EAESEDSV---------------EYDEQDK--------EH-SIETKKDK--------    374
Drosophila     KRRDLSAEQELTVLGMELTASCEGGHKWTGRPPTPVFRAPSEHSKLEDNLPEPQSIYLEG    428
C elegans      ------EEENSSELSEIEEE-EEEV--------KPVKKSKSSSQSVGKRKNRPKSTS---    329
zebrafish      ---ILTAEELAERMEKEEEV-EEEETA-----DDTCANEELNLE-----DNEEDEEDEEQ    323
Xenopus        ---QPSAEQIAEQMGFNEDD-DQGDSA-ENVGKEPCVECDEQAHVTVSAENKVDGI-GEN    301
human          ---ELSAEEIAERMGMIEEE-ESDHLGWESLPTDPRAQEDN---SVTSCDPKPETC----    303
mouse          ------------------------------------------------------------    227

Apis           ---------------ALPPQCELLVTDSETQAQKA------EEDVEAPVVLSGKMTLSLI    443
Lsa-RIC-3      ------------------------------------------------------------    374
Drosophila     ---------------ALAHESQILVADSQIKREEVYDSELNGSAEEPAIILSSRMTLSLI    473
C elegans      --EEEDEGEEESRKVAEDAEEEGIDIDSEIRE----------HAEKEKK-----------    366
zebrafish      EEEDEEEEEDEEEDIAEDQ---PCLPQSFDEDDEGLEY-SDEREEKPSRR-------RQI    372
Xenopus        LEEDEDEEEDDPEVIAENA---GFVSDSCNEEEDSKETFMNSRDENESL---------GD    349
human          --SCCFHEDEDPAVLAENA---GFSADSYPEQEETTKEEWSQDFKDEGL---------GI    349
mouse          ------------------------------------------------------------    227

Apis           SLDQNAA----VRKY---------------------------------------------    454
Lsa-RIC-3      ------------------------------------------------------------    374
Drosophila     NLDANQQ----NGNAGKSAVESPLADDIEIIGHDEQ------------------------    505
C elegans      --------DKNVRRRRPKKT----------------------------------------    378
zebrafish      TFSD-HRHVFHYPKGGAVGCKYETEEEEEHEGEEEEEGNEEDENGESEECEEEEEEEEEE    431
Xenopus        TLGSNQDRMGTLRKRNTKGIVY--------------------------------------    371
human          STDK-AYTGSMLRKRNPQGLE---------------------------------------    369
mouse          ------------------------------------------------------------    227

Apis           ------------------------------------------------------------    454
Lsa-RIC-3      ------------------------------------------------------------    374
Drosophila     ------------------------------------------------------------    505
C elegans      ------------------------------------------------------------    378
zebrafish      EEEEQHDKEEHGEREEKEEEDPLMEAESLGFNDDVECDPDEQEVDLIDFLQTYQPEVTVI    491
Xenopus        ------------------------------------------------------------    371
human          ------------------------------------------------------------    369
mouse          ------------------------------------------------------------    227

Apis           --------------------------  454
Lsa-RIC-3      --------------------------  374
Drosophila     --------------------------  505
C elegans      --------------------------  378
zebrafish      SNKSESSKRQASGTLRMRHKKEKVKK  517
Xenopus        --------------------------  371
human          --------------------------  369
mouse          --------------------------  227
```

Fig S3a: **Protein sequence alignment of *L. salmonis* RIC-3 with other RIC-3 proteins**. Protein sequences from, *Apis mellifera* (AJE70270.1), *L. salmonis*, *Drosophila melanogaster* (NP_611537.2),

*Caenorhabditis elegans (*NP_501299.1), *Danio rerio* (XP_017212528.2), *Xenopus laevis* (NP_001159914.1), *Homo sapiens* (AAP92162.1) and *Mus musculus* (XP_017177791.1). The two transmembrane domains (single line) and the coiled-coil domain are unlined (double line).

```
B.malayi-unc-50      ---------------------MVHAGTSFTFASSGGRGGRFPGSGYISPGARSITSNGSA    39
Cel-unc-50           MSSQ---PRGSGTQPGP--SQSPISQRNFRY--------EPARSGYTSPGQYSTYSTSTA    47
Hco-unc-50           MHNSSRNSTPAAYQPGTSGIQSPTLDTQFRF------GSSISRGGTATPGGYSTYST--S    52
Lsa-Unc-50           MSSTS-----------------------------TYKGPPYLS-------TGISSSSSSS    24
zebrafish-unc-50     ----------------------------------------------MLPT--SSPQIHRN    12
Xenopus-unc-50       ----------------------------------------------MLPTTSVSPRSPDN    14
Human-unc-50         MFLQN-----------------------------TCKRNVFLPSRKMLPSTSVNSLVQGN    31
Mouse-unc-50         ----------------------------------------------MLPSTSLSSSMHGN    14

B.malayi-unc-50      DHIGCFTAVKMTAIAKLNRYFRRLIRFRQMDFEFALWQMIYLLIKPQKVYRNFMYRKRTK    99
Cel-unc-50           DRVGCLTAVRMSAFAKLSRFTRRLVHIRQMDFEFALWQMLYLLIQPSKVYKNFIYRKRTK    107
Hco-unc-50           DRVGCFTAVRMSAWAKLTRFARRFVHFKQMDFEFALWQMLYLLIQPQKVYRNFIYRKRTK    112
Lsa-Unc-50           LPLPANSKTCMSAAAKRAKYLRRLLHFRQMDFEFAIWQMIYLLLDPKRVYKNFKYRKLSK    84
zebrafish-unc-50     GSLSERDAARHTAGAKRYKYLRRLLHFRQMDFEFAVWQMLYLFTSPQKVYRNFHYRKQTK    72
Xenopus-unc-50       GILSPRDATRHTAGAKRYKYLRRLFHFKQMDFEFALWQMLYLFTSPQKVYRNFHYRKQTK    74
Human-unc-50         GVLNSRDAARHTAGAKRYKYLRRLFRFRQMDFEFAAWQMLYLFTSPQRVYRNFHYRKQTK    91
Mouse-unc-50         GVLNSRDAARHTAGAKRYKYLRRLFRFRQMDFEFAAWQMLYLFTSPQRVYRNFHYRKQTK    74

B.malayi-unc-50      DQWARDDPAFLVLLLTALAVSSILFAWTIRLSFIGFIAFFLWAVFIDCICVGILIATVLW    159
Cel-unc-50           DQFARDDPAFLVLLALSLLFSSIFYAYALGLEKIGFFTFFLWSVFVDCIGVGVVIATVLW    167
Hco-unc-50           DQFARDDPAFLVLLSLSLIFSSVFYAVALGLTTWGFVKFFLWVIFIDCIGVGLIVATILW    172
Lsa-Unc-50           SQFARDDPAFLVLLSTWLVVSTAIYSWVLGIHFLGFIKFLLWVIGIDTLGAGAAVATVLW    144
zebrafish-unc-50     DQWARDDPAFLVLLSIWLCVSTVGFGLVLDMGFVETLTLLLWVVFIDCIGVGLLISTLMW    132
Xenopus-unc-50       DQWARDDPAFLVLLGIWLCVSTVGFGFVLDMSFFETFTLLLWVVFIDCVGVGLLIATSMW    134
Human-unc-50         DQWARDDPAFLVLLSIWLCVSTIGFGFVLDMGFFETIKLLLWVVLIDCVGVGLLIATLMW    151
Mouse-unc-50         DQWARDDPAFLVLLSIWLCVSTIGFGFVLDMGFFETIKLLLWVVFIDCVGVGLLISTLMW    134
                                      TM1                              TM2

B.malayi-unc-50      FASNRFLRR--VDDQDVEWGYCFDVHLNAFFPMLMLLHVLLPLTFSHLI--GFDAFLPRL    215
Cel-unc-50           WVSNRFLRK--VRDQDVEWGYCFDVHLNAFFPMLILLHVIVPILYPTLI--DSPAFLSIL    223
Hco-unc-50           WSSNQFLRK--VQDQDVEWGYCFDVHLNAFFPMLILLHVLLPIIYPTLV--DSPFFLPTA    228
Lsa-Unc-50           AFSNKYLLVRRD-DEAVEWGYCLDIHLNALFPVLVILHGIQLMFYHHFI--SGPGILSTI    201
zebrafish-unc-50     FVTNKYLMKHPNRDYDVEWGYAFDVHLNAFYPLLVILHFLQLFFINHVVVISSDWFLGYF    192
Xenopus-unc-50       FVSNKYMVNRQGKDYDVEWGYTFDVHLNAFYPLLVILHFIQLFFINHVI--LTGWFIGCF    192
Human-unc-50         FISNKYLVKRQSRDYDVEWGYAFDVHLNAFYPLLVILHFIQLFFINHVI--LTDTFIGYL    209
Mouse-unc-50         FVSNKYLVKRQSRDYDVEWGYAFDVHLNAFYPLLVILHFIQLFFINHVI--LTDTFIGYL    192
                                                           TM3

B.malayi-unc-50      FGNTIWFVAVVYYIYITFLGYTALPILKNTHIFLYPISFLFIFYVATVTAGWNISLTAMD    275
Cel-unc-50           LGNTFWFLAACYYVYITFLGYTALPILHKTQYFLYPISFIFMFFVATLTGGWNISRTALN    283
Hco-unc-50           LGNTIWFIAAVYYVYITFLGYTALPILHRTQYFLYPMTFLFISWVATITAGWNISRSAMG    288
Lsa-Unc-50           FGNTLWLIAITYYVFITFLGYGSLPGLKKTRVFLYPLGMLILLYFLSLFTGVNLCQILVK    261
zebrafish-unc-50     VGNTMWLIAIGYYVYITFLGYSALPFLKNTVVLLYPFALLGLLYVLSISLGWNFTKGLCW    252
Xenopus-unc-50       VGNTLWLIAIGYYIYITFLGYSALPFLKNTVVLLYPFAALALLYILSLALGWNFTAKLCL    252
Human-unc-50         VGNTLWLVAVGYYIYVTFLGYSALPFLKNTVILLYPFAPLILLYGLSLALGWNFTHTLCS    269
Mouse-unc-50         VGNTLWLIAVGYYIYVTFLGYSALPFLKNTVILLYPFAPLMVLYGLSLALGWNFTHTLCS    252
                              TM4                             TM5

B.malayi-unc-50      FYHLRAENRHRGS-----    288
Cel-unc-50           FYHSRAEPHKFAPQHGGL    301
Hco-unc-50           FYHYRVRTDD--------    298
Lsa-Unc-50           FYKDRVV-----------    268
zebrafish-unc-50     FYKHRVQ-----------    259
Xenopus-unc-50       FYKYRVR-----------    259
Human-unc-50         FYKYRVK-----------    276
Mouse-unc-50         FYKYRVK-----------    259
```

Fig S3b: **Protein sequence alignment of *L. salmonis* UNC-50 with other UNC-50 proteins**. Protein sequences from, *Brugia malayi* (CDP93422.1), *Caenorhabditis elegans (*CAA88132.2), *Haemonchus contortus (*CDJ98119.1), *L. salmonis*, *Danio rerio* (NP_956541.1), *Xenopus laevis* (NP_001088522.1), *Homo sapiens* (NP_001317283.1) and *Mus musculus* (NP_001343457.1). Five transmembrane domains are underlined.

```
Lsa-Unc-74      ------MQSIP--LVYIFICLSIHAAYGKVLELSDRFIPLKHEGMWLIKFYAPWCGHCKK      52
Cel-unc-74      MQKYFLLPLLSLSVLLFVYDTEATNPPTAVLDLSDKFLDVKDEGMWFVEFYAPWCAHCKR      60
Hco-unc-74      ------MQFYPLILLSIVPCCFAAGTLTVVIDLNEKFLDVMNEGFWIVKFYAPWCAHCKR      54
Oden-unc-74     ------MRFSPLSLLAVVAICSAAGPLTVVIDLNEKFLDVSDEGLWIVKFYAPWCAHCKR      54
                                                      Thioredoxin domain

Lsa-Unc-74      LEPIWKLSEQSLA--HDPVRVGRVDCTRFPIVATEFNIQGFPTILFLKGENQYTYEGDRT      110
Cel-unc-74      LHPVWDQVGHTLSDSNLPIRVGKLDCTRFPAVANKLSIQGYPTILFFRNGHVIDYRGGRE      120
Hco-unc-74      LFPIWEHLGHAVNDKSLPVRVAKMDCTRFTSVCNSLSISGYPTILFFRQGRRLEYTGERS      114
Oden-unc-74     LLPVWEHLGYAVSDKNLPVRVAKMDCTRFTSACNKLSISGYPTVIFFRNGRRIEYHGERT      114
                                 Thioredoxin domain

Lsa-Unc-74      REDIVAFSKKLLGPSVTQIKTEGD-FRKAMERSEIFFVYSGSSEGLLYKHFKSLAAAHQQ      169
Cel-unc-74      KEALVSFAKRCAAPIIEVINENQIEKVKLSARSQPSYVFFGTSSGPLFDAFNEAASSKFS      180
Hco-unc-74      KEDLFNFVVKSSAPIIEKINAVRVNDIRRDSRSDPSFVIIGDEKDGTYTKFEEIAQSLFS      174
Oden-unc-74     KEALFNFVVKSSAPIVEKVNAVRLNEIRRDSRNDPAFVIIGEDKDEMQAEFEAVADSLFS      174

Lsa-Unc-74      YDFFYHA-PPEIVQKFSDVSHKKNVIRVFKDGTSHKFEDNGAFEDPPKAESLTMEKYKAE      228
Cel-unc-74      VARFYSVAPPENDASFR------QRVAVFKDNF--------------------------      207
Hco-unc-74      KTRFFSASPASVPATLRESG---ARVAVFKDDS--------------------------      204
Oden-unc-74     KTRFFSASPAAVPTSLRESG---SRIAVFKDDS--------------------------      204

Lsa-Unc-74      EDEKESVFVTLDPRNVSLVSWVNSERFPLFMKVTRGKLNHLMLSKKLLVMAVLEENRIGE      288
Cel-unc-74      -------EIEFNGDIEKLTEWVTRERWPGFLQATSSNLAEIGASGKLVVLVVSSESHKFN      260
Hco-unc-74      -------FFPYHGSVEGLESWIMAERWPLMPRASGVNLRDLATTKKLLVLVISNELERYN      257
Oden-unc-74     -------FFPYHGTVEGLKRWIMAERWSLMPRATPTNIAEIGTNGKLTVLVVCTELDRLN      257

Lsa-Unc-74      LSEEMLEFKEMIKLVLE--RNLHRYRSIFQFGWIGSPELANSVAMETLSLPNLLVLNVSS      346
Cel-unc-74      NTSPIREFHKTAEEASKELRKHPDLWNRFQFAWLDGSDLASQIQMAAVSEPHLFIFNYTS      320
Hco-unc-74      QSTPVGRFYEKARGAAEDLRKSQYLWSRYQFAWMDGSDIVSTIVMNNMEAPNIIVLNYTT      317
Oden-unc-74     QTSPIGIFCEMARGAAEELRKNEYLWSRFQFAWLDGPEIAMNIVMGNMDPPNILVFNYST      317

Lsa-Unc-74      YQHHLPDDDPSFMTPEAVQLFLEDIVQ----GSAPVYGGSSYNVRLYRAYYELRSNLFEM      402
Cel-unc-74      YEYYLSEDEPSQMTIKSILTFLEQTSEGIDKETIVAFGGRHLLTRIKRMAFELYWNIAQM      380
Hco-unc-74      YEYFLNEDDPEKVTRGSLVTWLSNLADGTEKGTATPLGGRSLLTRIRRIIFELYMNVTQM      377
Oden-unc-74     YEYYLSDDEPEKMTKASIVTWLNHLADALEKKVAVGHGGRSWPTRIKRMIYELYTNVAQM      377

Lsa-Unc-74      WKGNPALTAVLFGLPFGFFSLICYSICCADIMDAEEE--------DEDHETEAHE---KV      451
Cel-unc-74      FATQPLLSSCLFGVPIAFLSIICYSICSADFTVDRDEFYGDEDELIDDEEGEETEHPETD      440
Hco-unc-74      FATQPLLSSVLFGMPIAFLSIICYSICSADFSVDREEFYPDDEEEEEVEAGEESESALLV      437
Oden-unc-74     FSTQPLLSSCLFGVPIAFLSIICYSICSADFTVDREEFYPDDEDEDYDEAVEEDERAHLV      437

Lsa-Unc-74      D-------    452   TM
Cel-unc-74      -DDHEKAE    447
Hco-unc-74      DQNHVKNE    445
Oden-unc-74     DASHAKNE    445
```

Fig S3c: Protein sequence alignment of *L. salmonis* UNC-74 with other UNC-74 proteins. Protein sequences from L. salmonis, *Caenorhabditis elegans* (NP_491361.1) and *Haemonchus contortus* (ADV92278.1) and *Oesophagostomum dentatum* (JAA65034.1). C-terminal transmembrane domain and the Thioredoxin domain are underlined. Only these sequences were available in Genbank. This protein is uncharacterized in majority of species unlike other Unc proteins.
