# Supplementary material for: Nicotinic acetylcholine receptors: Ex-vivo expression of functional, non-hybrid, heteropentameric receptors from a marine arthropod, Lepeophtheirus salmonis
Source: PLoS Pathog. 2020 Jul 27;16(7):e1008715. doi: 10.1371/journal.ppat.1008715 (PMC7419010; doi:10.1371/journal.ppat.1008715)
Supplement: S4 Fig — The first trace corresponds to an acetylcholine control pulse (100 μM) that serves as the maximal peak current. The different concentrations tested (in nM) are indicated above each individual trace. (PDF) [file ppat.1008715.s005.pdf]

<table>
<tr><th>Compounds</th><th>Lsa-nAChR-1</th><th>Lsa-nAChR-2</th></tr>
<tr><td>Nicotine</td><td>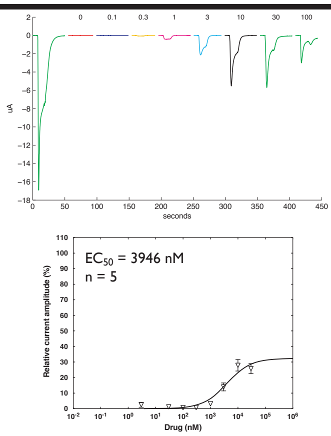
</td><td>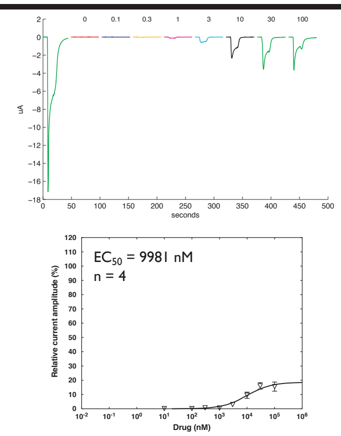
</td></tr>
<tr><td>Acetamiprid</td><td>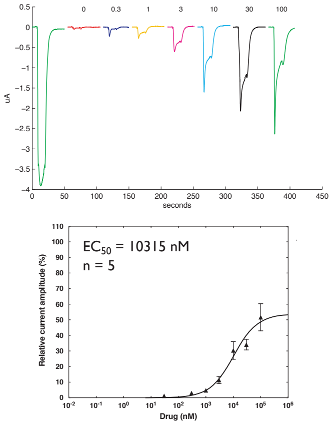
</td><td>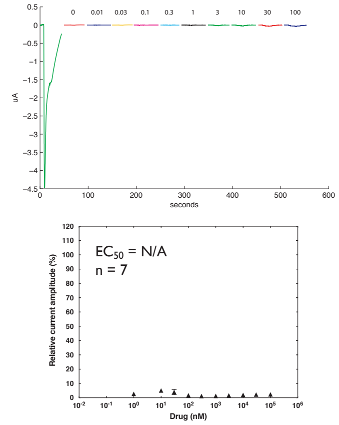
</td></tr>
<tr><td>Clothianidin</td><td>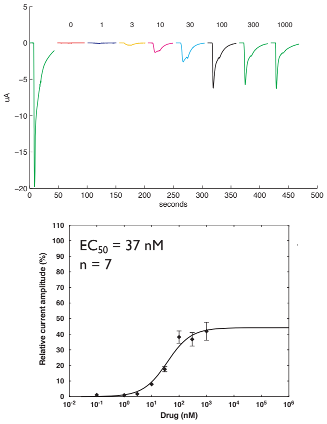
</td><td>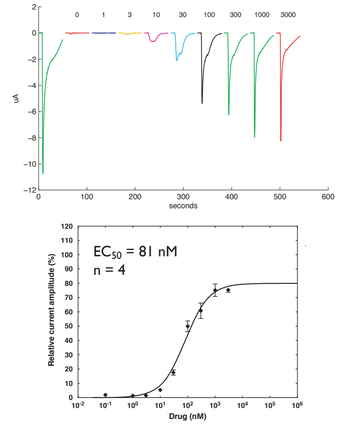
</td></tr>
<tr><td>Dinotefuran</td><td>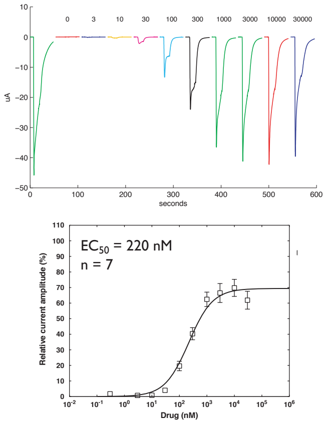
</td><td>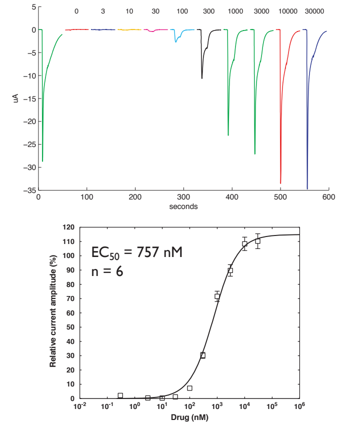
</td></tr>
</table>

Compounds	Lsa-nAChR-1	Lsa-nAChR-2
**Imidacloprid**	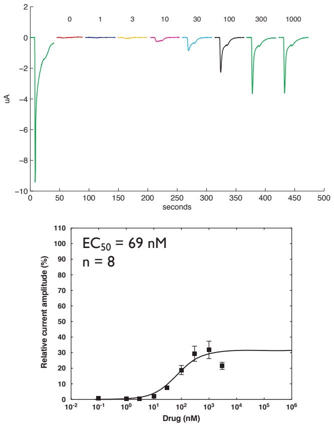	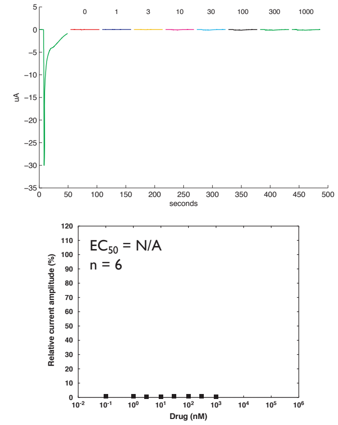
**Nitenpyram**	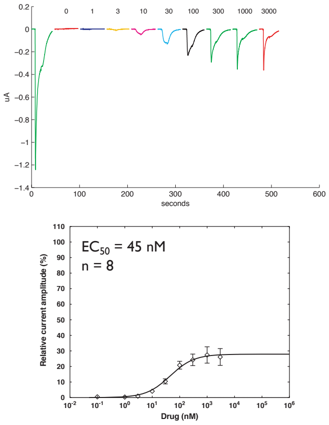	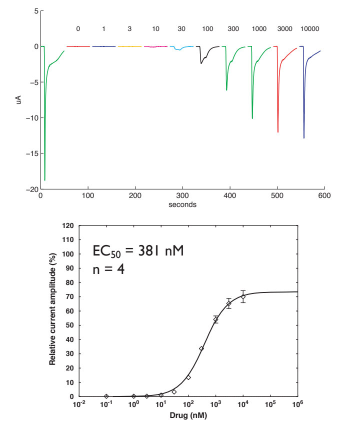
**Thiacloprid**	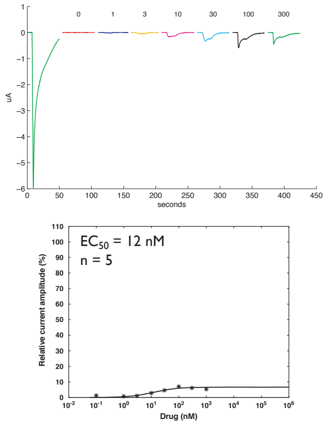	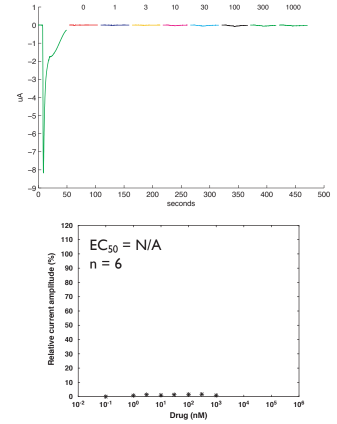

Fig S4: Typical current traces obtained from Lsa-nAChR-1 or Lsa-nAChR-2 upon exposition to selected compounds. The first trace corresponds to an acetylcholine control pulse (100 µM) that serves as the maximal peak current. The different concentrations tested (in nM) are indicated above each individual trace.
