# Supplementary material for: Nicotinic acetylcholine receptors: Ex-vivo expression of functional, non-hybrid, heteropentameric receptors from a marine arthropod, Lepeophtheirus salmonis
Source: PLoS Pathog. 2020 Jul 27;16(7):e1008715. doi: 10.1371/journal.ppat.1008715 (PMC7419010; doi:10.1371/journal.ppat.1008715)
Supplement: S1 Table — Counts for each of the subunits from an RNaseq study of 16 individual adult L. salmonis females are provided. The transcriptome file was generated from the predicted L. salmonis transcriptome (ftp://ftp.ensemblgenomes.org/pub/metazoa/release-47/fasta/lepeophtheirus_salmonis/cds/). Assembly files (bam-format) for reads mapping to the transcriptome file in the folder S1 Data. (PDF) [file ppat.1008715.s006.pdf]

Table S1: The nAChR subunits identified in *L.salmonis* genome: six were α subunits and two were β subunits. Counts for each of the subunits from an RNaseq study of 16 individual adult *L. salmonis* females are provided. The transcriptome file was generated from the predicted *L. salmonis* transcriptome (ftp://ftp.ensemblgenomes.org/pub/metazoa/release-47/fasta/lepeophtheirus_salmonis/cds/). Assembly files (bam-format) for reads mapping to the transcriptome file in the folder Table_S1_bam.

**COUNTS (fragments)**								
**ENSEMBL ID**	**EMLSAT00000009264**	**EMLSAT00000000084**	**EMLSAT00000001002**	**EMLSAT00000005313**	**EMLSAT00000003136**	**EMLSAT00000007472**	**EMLSAT00000007263**	**EMLSAT00000006919**
**Individual**	**Lsa_nAChRα1**	**Lsa_nAChRα2**	**Lsa_nAChRα3**	**Lsa_nAChRα4 ***	**Lsa_nAChRα7**	**Lsa_nAChRα6 ***	**Lsa_nAChRβ1**	**Lsa_nAChRβ2**
1	84	28	23	2	2	4	45	34
2	110	47	23	5	3	8	40	39
3	114	19	30	2	4	23	46	44
4	50	14	13	0	3	5	21	23
5	92	33	39	1	1	7	35	33
6	118	51	36	0	2	18	43	40
7	128	56	21	6	3	8	44	80
8	57	22	14	4	2	27	26	33
9	104	43	27	4	1	26	26	38
10	131	49	23	6	1	16	51	35
11	89	43	18	1	1	27	46	34
12	128	30	35	0	3	5	10	41
13	99	18	42	0	5	21	36	48
14	99	31	28	3	1	7	30	54
15	90	23	46	0	1	9	11	75
16	80	33	19	1	1	13	30	46
MEDIAN	99	32	25	2	2	11	36	40
* the nAChR subunits for which the full-length cDNA sequence could not be identified								
