# Supplementary material for: Nicotinic acetylcholine receptors: Ex-vivo expression of functional, non-hybrid, heteropentameric receptors from a marine arthropod, Lepeophtheirus salmonis
Source: PLoS Pathog. 2020 Jul 27;16(7):e1008715. doi: 10.1371/journal.ppat.1008715 (PMC7419010; doi:10.1371/journal.ppat.1008715)
Supplement: S2 Table — (PDF) [file ppat.1008715.s007.pdf]

Table S2: The putative open reading frame and protein length of *L.salmonis* nAChR subunits and ancillary proteins, along with their GenBank accession numbers.

Gene name	ORF length(in bp)	Protein length (in aa)	GenBank accession numbers
Lsa-nAChR α1	1779	595	MN178318
Lsa-nAChR α2	1581	526	MN178319
Lsa-nAChR α3	1692	561	MN178320
Lsa-nAChR α7	1455	484	MN178321
Lsa-nAChR β1	1920	639	MN178322
Lsa-nAChR β2	1536	511	MN178323
Lsa-unc-74	1359	452	MN240314
Lsa-unc-50	807	268	MN240313
Lsa-ric3	1122	374	MN240312
