# Supplementary material for: Nicotinic acetylcholine receptors: Ex-vivo expression of functional, non-hybrid, heteropentameric receptors from a marine arthropod, Lepeophtheirus salmonis
Source: PLoS Pathog. 2020 Jul 27;16(7):e1008715. doi: 10.1371/journal.ppat.1008715 (PMC7419010; doi:10.1371/journal.ppat.1008715)
Supplement: S3 Table — (PDF) [file ppat.1008715.s008.pdf]

Table S3. Primers used for PCR amplification of nAChR subunits and ancillary proteins from *Lepeophtherius salmonis*.

**Primer name**	**Sequence 5' → 3'**
Primers for full-length ORF amplification	
NheI_Lsa-nAChRa1_F1	GGCGGCTAGCAGATTTCGCATCCTCTCCAA
XhoI_Lsa-nAChRa1_R1	GGCGCTCGAGTTTTGGCTTCTTCTTCTTCTTCA
NheI_Lsa-nAChRa2F1	GGCGGCTAGCAAGAAGGGATCGAAAATGCTT
XhoI_Lsa-nAChRa2R1	GGCGCTCGAGTGAGCAAGAGGATGTTTTCATT
NheI_Lsa-nAChRa3F1	GGCGGCTAGCGAAAACATGGACAAAGTTTGGA
XhoI_Lsa-nAChRa3R1	GGCGCTCGAGGGAGGGTGGGGTGTAGGTAT
NheI_Lsa-nAChRa7F1	GGCGGCTAGCTGCAACGGTCTATCAGAATGA
XhoI_Lsa-nAChRa7R1	GGCGCTCGAGTGAATTTAGCACCTGTTTACCAAA
NheI_Lsa-nAChRb1_F1	GGCGGCTAGCCATCATCAAGAATGGATTGGAA
XhoI_Lsa-nAChRb1_R1	GGCGCTCGAGTGCAGCTGTATTTCCTTCTTCT
NheI_Lsa-nAChRb2F1	GGCGGCTAGCGCATAGCGTTTCAAATGTTTCTT
XhoI_Lsa-nAChRb2R1	GGCGCTCGAGGCAAATGGGTGGGATGATAC
NheI_Lsa-ric-3_F1	GGCGGCTAGCTATGGCAGACAAAAGCTTCAAT
NotI_Lsa-ric-3_R1	GGCGGCGGCCGCTCATTTATTTGTCTTTCTTTGTCTCT
NheI_Lsa-unc-50_F1	GGCGGCTAGCAGACGAGGAACAGCGTGACT
XhoI_Lsa-unc-50_R1	GGCGCTCGAGAACCAGGTGAAATGAGAGCAA
NheI_Lsa-unc-74_F1	GGCGGCTAGCCATGCAATCGATACCTCTGG
XhoI_Lsa-unc74_R1	GGCGCTCGAGGGACTAGTCAACTTTTTCATGTGC

Primers for 5'- and 3'-RACE PCR	
Lsa-nAChRa2_F1	TCAGCCGGATAATGACACTG
Lsa-nAChRa2_F2	ATGCTGCCCAGAACCCTAC
Lsa-nAChRa2_R3	TCTTCGAACCCATTGGGGCATTC
Lsa-nAChRa2_R4	TTTCCCCAGACTGAGCAGGCAAA
Lsa-nAChRa3_F1	GGTCAAAGCCCAGAATCAAA
Lsa-nAChRa3_F2	ACCCCTGAGAATCTCCCATC
Lsa-nAChRa7_F1	AATGCCTGCAATTCGAACA
Lsa-nAChRa7_F2	TTCAACTACGACGTCGAACACT
Lsa-nAChRa7_R5	CCATTGCTGCCAATTTCCATTCG
Lsa-nAChRa7_R6	CAGAGGCAAGATTCCCTTGGTTTGG
Lsa-nAChRb2_F1	TGGGGATTTCTTTCTTGACG
Lsa-nAChRb2_F2	CTCCGACATCTTTGGCAGTT
Lsa-nAChRb2_R3	CAGCTCTGTTCGTCGAATGGAAACCA
Lsa-nAChRb2_R4	GCTGGAGGCTTCCACACTACTTCACCA
Lsa-unc-74_F1	CCGCGGCACACCAACAGTATGACTT
Lsa-unc-74_F2	TCATGCTCCACCCGAAATTGTTCAAA
Lsa-unc-74_R1	CCATCGTGAGGGATTCGGCTTTTG
Lsa-unc-74_R2	TTGAACAATTTCGGGTGGAGCATGA
